# Concentration and Length Dependence of DNA Looping in Transcriptional Regulation

**DOI:** 10.1371/journal.pone.0005621

**Published:** 2009-05-25

**Authors:** Lin Han, Hernan G. Garcia, Seth Blumberg, Kevin B. Towles, John F. Beausang, Philip C. Nelson, Rob Phillips

**Affiliations:** 1 Department of Applied Physics, California Institute of Technology, Pasadena, California, United States of America; 2 Department of Physics, California Institute of Technology, Pasadena, California, United States of America; 3 Department of Physics and Astronomy, University of Pennsylvania, Philadelphia, Pennsylvania, United States of America; German Cancer Research Center, Germany

## Abstract

In many cases, transcriptional regulation involves the binding of transcription factors at sites on the DNA that are not immediately adjacent to the promoter of interest. This action at a distance is often mediated by the formation of DNA loops: Binding at two or more sites on the DNA results in the formation of a loop, which can bring the transcription factor into the immediate neighborhood of the relevant promoter. These processes are important in settings ranging from the historic bacterial examples (bacterial metabolism and the lytic-lysogeny decision in bacteriophage), to the modern concept of gene regulation to regulatory processes central to pattern formation during development of multicellular organisms. Though there have been a variety of insights into the combinatorial aspects of transcriptional control, the mechanism of DNA looping as an agent of combinatorial control in both prokaryotes and eukaryotes remains unclear. We use single-molecule techniques to dissect DNA looping in the *lac* operon. In particular, we measure the propensity for DNA looping by the Lac repressor as a function of the concentration of repressor protein and as a function of the distance between repressor binding sites. As with earlier single-molecule studies, we find (at least) two distinct looped states and demonstrate that the presence of these two states depends both upon the concentration of repressor protein and the distance between the two repressor binding sites. We find that loops form even at interoperator spacings considerably shorter than the DNA persistence length, without the intervention of any other proteins to prebend the DNA. The concentration measurements also permit us to use a simple statistical mechanical model of DNA loop formation to determine the free energy of DNA looping, or equivalently, the 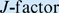 for looping.

## Introduction

The biological significance of DNA is primarily attributed to the information implicit in its sequence. Still, there are a wide range of processes for which DNA's physical basis as a stiff polymer also matters [1]. For example, the packaging of DNA into nucleosomes appears to select for sequence motifs that are particularly flexible [2], [3]. In the setting of transcriptional regulation, there are a host of regulatory architectures both in prokaryotes and eukaryotes which require the interaction of sequences on the DNA that are not adjacent [4]–[7]. These interactions are mediated by DNA-binding proteins, which have to deform the DNA. In eukaryotes, action of transcription factors over long distances seems the rule rather than the exception. One of the most transparent examples of DNA looping is in bacteria where some repressors and activators can bind at two sites simultaneously, resulting in a DNA loop. This effect was first elucidated in the context of the arabinose operon [8]. It is an amusing twist of history that the two regulatory motifs considered by Jacob and Monod, namely, the switch that makes the decision between the lytic and lysogenic pathways after phage infection [9] and the decision making apparatus associated with lactose digestion in bacteria [5], [10], both involve DNA looping as well.

To understand the physical mechanism of the biological action at a distance revealed by DNA looping, it is necessary to bring both *in vitro* and *in vivo* experiments as well as theoretical analyses to bear on this important problem. Over the last few decades there have been a series of impressive and beautiful experiments from many quarters that inspired our own work. In the *in vivo* context, it is especially the work of Müller-Hill and coworkers that demonstrates the intriguing quantitative implications of DNA looping for regulation [11]. In their experiments, they tuned the length of the DNA loop in one base pair increments and measured the resulting repression. More recently, these experiments have been performed with mutant bacterial strains that were deficient in architectural proteins such as HU, IHF and H-NS [12], [13]. On the *in vitro* side, single molecule experiments using the tethered-particle method [14]–[22] have also contributed significantly [23]–[28]. The idea of these experiments is to tether a piece of DNA to a microscope coverslip with a bead attached to the end. The DNA construct has the relevant binding sites (operators) for the protein of interest along the DNA and when one of these proteins binds, it shortens the length of the tether. As a result of the shorter tether, the Brownian motion of the bead is reduced. Hence, the size of the random excursions of the bead serves as a reporter for the status of the DNA molecule (i.e. looped or unlooped, DNA-binding protein present or not).

In addition to single-molecule studies, *in vitro* biochemical assays have also shed important light on the interactions between transcription factors and their DNA targets. Both filter binding assays and electrophoretic mobility shift assays have been widely used to study how variables dictating DNA mechanics such as length and degree of supercoiling, alter the looping process [29]–[33].

One of the missing links in the experimental elucidation of these problems is systematic, single-molecule experiments which probe the length, repressor concentration and sequence dependence of DNA looping. Such experiments will complement earlier *in vivo* work, which has already demonstrated how DNA length and repressor concentration alter repression [11]. Our view is that such systematic experiments will help clarify the way in which both length and sequence contribute to the probability of DNA looping, and begin to elucidate the mechanisms whereby transcription factors act over long genomic distances. Further, such experiments can begin to shed light on broader questions of regulatory architecture and the significance of operator placement to transcriptional control. To that end, we have carried out experiments that probe the DNA looping process over a range of concentrations of repressor protein and for a series of different loop lengths. In addition, intrigued by the sequence preferences observed in nucleosomal DNA, we have made looping constructs in which these highly bendable nucleosomal sequences are taken out of their natural eukaryotic context and are inserted between the operators that serve as binding sites for the Lac repressor (the results of those experiments will be reported elsewhere). The point of this exercise is to see how the looping probability depends upon these tunable parameters, namely, length, repressor concentration and sequence.

Our key results are: (1) The concentration dependence of looping as a function of repressor concentration (a “titration” curve) can be described by a simple equilibrium statistical-mechanics model of transcription factor-DNA interactions. The model predicts a saturation effect, which agrees with our experimental observations. (2) By measuring this effect, we were able to isolate the free energy change of looping (that is, separate it from the binding free energy change), obtaining an experimental measurement of its value for a range of different lengths in an uncluttered, *in vitro*, setting. (3) Systematic measurement of looping free energy as a function of interoperator spacing hints at the same modulations seen in analogous *in vitro* work on cyclization [3], [34], and *in vivo* work on repression [11], [12]. (4) Clear experimental signature of multiple looped states, consistent with theory expectations [35]–[38] and other recent experiments [26], [28]. In the remainder of the paper, we describe a series of experiments that examine both the length and concentration dependence of DNA looping induced by the Lac repressor. A companion paper gives extensive details about our theoretical calculations [39].

## Results

As argued above, one of our central concerns in performing these experiments was to have sufficient, systematic data to make it possible to carry out a thorough analysis of the interplay between theories of transcriptional regulation (and DNA looping) [40]–[43], and experiment. To that end, we have carried out a series of DNA looping experiments using the tethered-particle method [23] for loop lengths ranging from 300 to 310 bp in one base pair increments as well as several representative examples for lengths below 100 bp. The experiments described here use DNA constructs harboring two different operators, symmetric operator 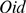 and primary natural operator 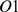 as Lac repressor binding sites. In addition, we have explored how the looping trajectories depend upon the concentration of Lac repressor. The particular experimental details are described in the “Materials and Methods” section.

**Figure 1:**
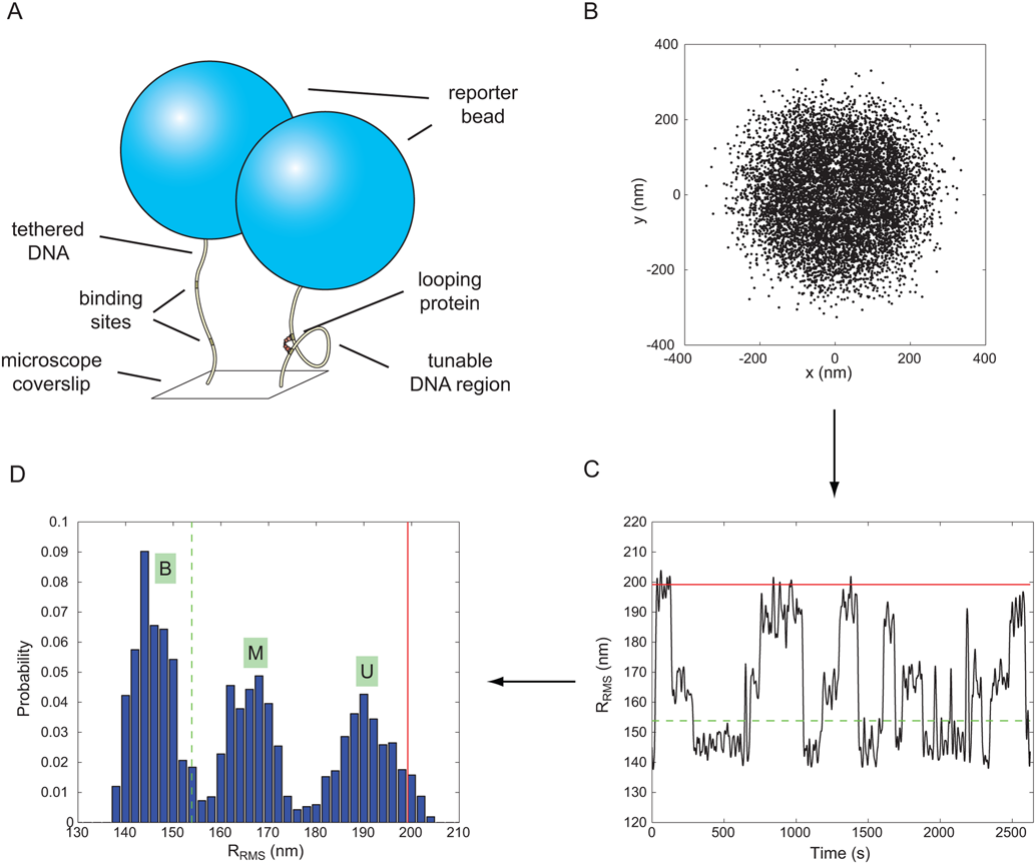
Different representations of TPM data. (A) Schematic of the TPM experiment. (B) Scatter plot of drift-corrected positional data. Each dot corresponds to the instantaneous projected position of the bead at a particular instant in time. (C) Running average of Gaussian filtered RMS motion over an effective window of 4 seconds. 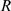 is the distance from the bead center (dots in panel (B)) to the tether attachment point (centroid of all dots in panel (B)). Red (solid) and green (dashed) lines represent naively expected motion, based on calibration measurements [91] in the absence of any DNA binding protein, for 901 bp DNA and an imagined DNA for which 305+20.5 bp (the center to center distance between operators) are subtracted off of the full length 901 bp tether. (Fig. 11 gives a more precise prediction of the expected excursions in looped states.) (D) Histogram of the RMS motion. Different peaks correspond to looped (labeled B, bottom, and M, middle) or unlooped (labeled U) states. The lines shown here are the same as those shown in (C). The presence of Lac repressor results in a shift of the excursion of the unlooped state with respect to the excursion expected from the protein-free calibration curve. This is reflected in the fact that the U peak does not coincide with the red line. The DNA used here is pUC305L1 (see Materials and Methods section) with 100 pM Lac repressor. A detailed discussion of how to go from microscopy images of beads to traces and histograms like those shown here is given in the movie S1 in the Supporting Information.

A typical experimental trace resulting from these measurements is shown in fig. 1. (Representative examples of experimental traces from all of the lengths and concentrations considered throughout the paper as well as examples of rejected traces are shown in the Supporting Information S1.) As seen in the figure, as with other recent work [26], [28], there are clearly two distinct looped states as seen both in the trajectory and the histogram. Control experiments with one of the two binding sites removed show only the highest peak, which further supports the idea that the two lower peaks indeed indicate looped configurations. One hypothesis is that these two looped states correspond to two different configurations of the Lac repressor molecule and its attendant DNA, which we will refer to as the “open” and “closed” configurations. Direct interconversion between the two looped species suggested the two distinct looped states are indeed due to different conformations of Lac repressor protein [26]. An alternate hypothesis is that the two peaks reflect different DNA topologies [44]–[46]. Although this hypothesis does not obviously accommodate the apparent observation of direct interconversion, nevertheless we will present data from Monte Carlo simulations of DNA chain conformations that show that it *can* quantitatively explain the observed multi-peak structure observed in the data.

### Concentration dependence

**Figure 2:**
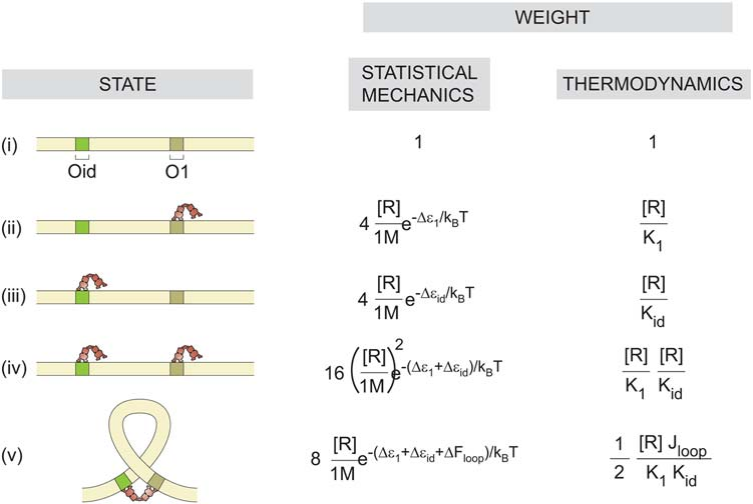
States and weights for the Lac repressor-DNA system [42]. Each of the five state classes shown in the left column has a corresponding statistical weight given by the product of the Boltzmann factor and the microscopic degeneracy of the state. All of the weights have been normalized by the weight of the state in which the DNA is unoccupied. State (v) is treated as a single looped state, even though there are multiple distinct looped configurations. The third column shows how to write these statistical weights in the language of equilibrium constants and 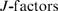. The derivation of these weights and the relation between the statistical mechanical and thermodynamic perspectives can be found in the Supporting Information S1.

In order to extract quantities such as the free energy of looping associated with repressor binding (or equivalently, a 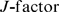 for looping, essentially the concentration at which in a solution of DNA with sticky ends, the probability of forming circles and dimers is equal) and to examine how the propensity for looping depends upon the number of repressors, we needed looping data at a number of different concentrations. At very low concentration, we expect that there will be negligible looping because neither of the operators will be bound by Lac repressor. At intermediate concentrations, the equilibrium will be dominated by states in which a single repressor tetramer is bound to the DNA at the strong operator, punctuated by transient looping events. In the very high concentration limit, each operator will be occupied by a tetramer (see fig. 2 below), making the formation of a loop nearly impossible.

**Figure 3:**
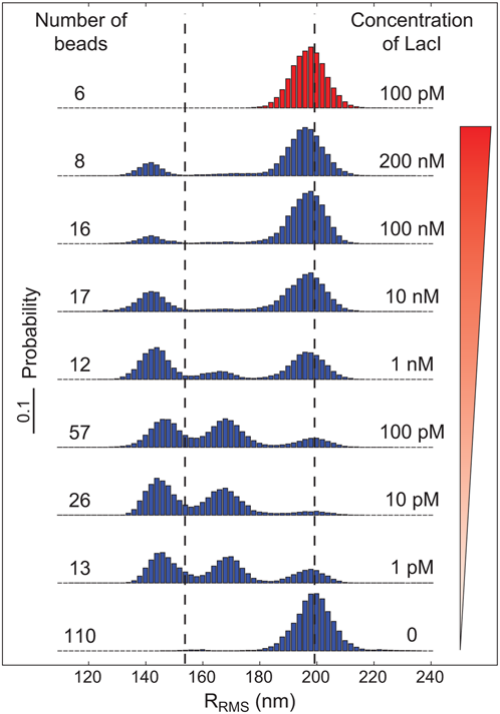
Concentration dependence of the distribution of bead excursions. The histograms show the distribution of RMS motions averaged over 4 seconds at different concentrations of Lac repressor. The blue histograms correspond to measurements for a length between operators of 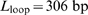 (see fig. 15), whereas the red histogram is a control where 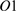 has been deleted. The two dashed lines represent the naively expected motion, based on our calibration measurements [91]. (See fig. 11 for a more precise prediction of the peak locations.)

This progression of qualitative behavior is indeed seen in fig. 3, which shows data from eight distinct concentrations of Lac repressor, as well as a single-operator control in which the DNA lacks a secondary operator. Throughout this work we define *sequence length* or *loop length* as the end-to-end distance between the operators as shown in fig. 15. These curves correspond to a sequence length of 306 bp and are generated by summing the normalized histograms from *all* of the individual trajectories for each concentration that pass our bead selection criteria (bead selection criteria are discussed in detail in the Supporting Information S1). A key feature of these data is the way in which the two looped states are turned off as the concentration of Lac repressor is increased to very high levels. This phenomenon is expected since the Lac repressor exists always as tetramers under the conditions used here [47], [48], and competition for binding at the second operator between loose Lac repressor and Lac repressor bound to the other operator is stronger as the concentration of Lac repressor increases. However, the two different looped species have slightly different responses at high repressor concentrations. For example, at 1 nM concentrations, the intermediate looped state has become very infrequent, whereas the shortest looped state remains competitive. Similar concentration dependence of Lac repressor mediated DNA looping was studied previously [24] at 4 pM, 20 pM and 100 pM. Those experiments revealed that looping is suppressed as the concentration goes up.

**Figure 4:**
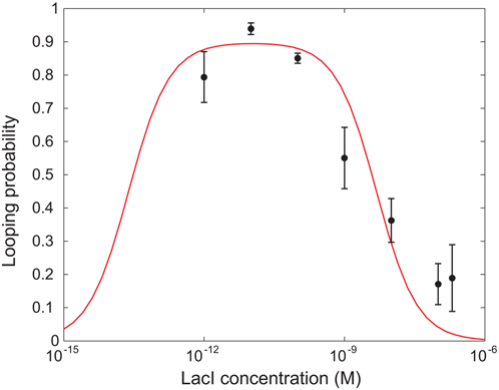
Looping probability 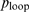, at different concentrations of Lac repressor. The DNA used in these experiments is 901 bp long and the loop length is 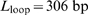. The vertical axis gives looping probability (fraction of time spent in either of the two looped states). The fraction of time spent in the looped states was calculated for each bead individually and the mean and standard error calculated for each construct. The curve is a fit to the experimental data using the statistical mechanics model described in the text. The obtained parameters are shown in table 1 under “Nonlinear fit”.

One way to characterize the looping probability as a function of concentration is shown in fig. 4. There are various ways to obtain data of the sort displayed in this plot. First, by examining the trajectories, we can simply compute the fraction of time that the DNA spends in each of the different states, with the looping probability given by the ratio of the time spent in either of the looped states to the total elapsed time. Of course, to compute the time spent in each state, we have to make a thresholding decision about when each transition has occurred. This can be ambiguous, because trajectories sometimes undergo rapid jumps back and forth between different states; it is not unequivocally clear when an apparent transition is real, and when it is a random fluctuation without change of looping state. A second way of obtaining the looping probability is to use fig. 3 and to compute the areas under the different peaks and to use the ratios of areas as a measure of looping probability. This method, however, does not properly account for possible variation between different beads, because they are all added up into one histogram. A third alternative is to obtain the looping probability for each *individual* bead, by plotting its histogram and calculating the area under that subset of the histogram corresponding to the looped states. We used this last method to calculate the mean looping probability and the standard error for each construct, which is shown in fig. 4.

These results can also be explored from a theoretical perspective using the tools of statistical mechanics [42], [43], [49]. The goal of a statistical mechanical description of this system is to compute the probability of the various microstates available to the repressor-DNA system as shown in fig. 2. The simplest model posits 5 distinct states [23], [24], [26]: Both operators empty, 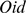 occupied by repressor without looping, 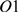 occupied by repressor without looping, 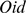 and 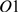 separately occupied by single repressors and the looped state (the subtleties associated with the statistical weight of the looped state are described in the Supporting Information S1). The model does not take into account the effect of non specific binding of Lac repressor to non-operator DNA, because a simple estimate reveals that the vast majority of repressors are free in solution rather than bound nonspecifically to the tethered DNA. We argue that this effect is negligible because the equilibrium association constant of Lac repressor to non-operator DNA at conditions similar to ours is around 10^6^∼10^7^ M^−1^ [50]–[56], which is roughly six orders of magnitude less than the corresponding quantity for specific binding [30], [57]–[62]. Given such association constants, the ratio between non specifically bound Lac repressor and the free Lac repressor in solution is given as
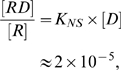
where 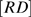 is the concentration of non-specifically bound Lac repressor, 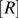 is the concentration of Lac repressor in solution, and 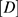 is the DNA concentration, which is around 2 pM in our experiment. For 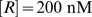, we have 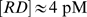, which is far smaller than the concentration of Lac repressor in solution.

**Figure 5:**
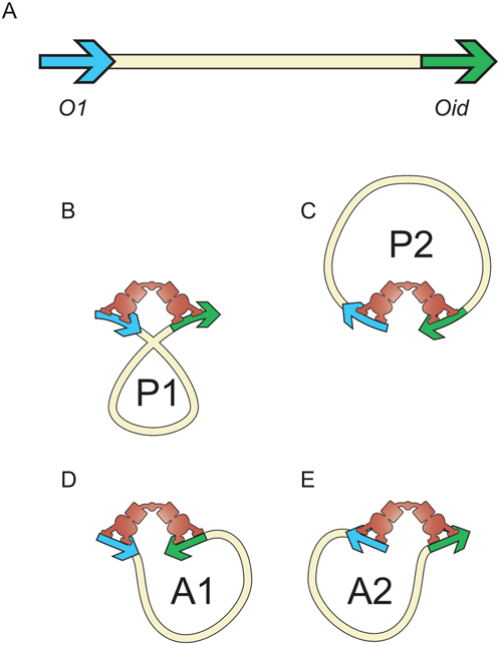
Schematic showing the different looping topologies associated with binding of Lac repressor. (A) Orientation of the two operators on the DNA. Choice of labeling orientation is arbitrary. (B)–(E) two parallel (P1 and P2) and antiparallel (A1 and A2) orientations of the DNA when subjected to Lac repressor mediated looping. We adopted the naming conventions given in refs. [36], [37].

It is convenient to describe the probability of the various states using both the language of microscopic binding energies (and looping free energies) and the language of equilibrium constants (and 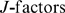). From a microscopic perspective, the key parameters that show up in the model are the standard free energy changes for repressor binding to the two operators, 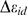 and 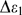, the looping free energy 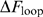 and the concentration of repressor 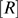. The binding energy here contains two components. One is the standard positional free energy required for bringing one Lac repressor molecule to its DNA binding site at 1 M concentration of Lac repressor. The other is the rotational entropy loss times 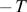, plus the interaction free energy due to the physical contact upon protein binding [42], [43], [63]. The associated free energy with each configuration gives the statistical weights of the equilibrium probability (listed in the middle column of fig. 2). For example, to obtain the probability of the looped state, we construct the ratio of state (v) in the figure to the sum over all five states, as given by
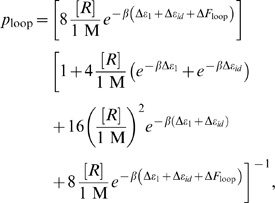
(1)where 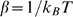 and the temperature is in degrees Kelvin. As detailed in the Supporting Information S1 and can be read off from the right column in fig. 2, this microscopic description is conveniently rewritten in terms of the equilibrium constants and 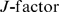 for looping as
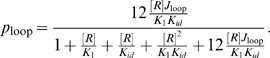
(2)Here 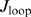 is the average of the individual 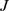 factors corresponding to different loop topologies. These topologies can be classified according to the orientation of each one of the operators with respect to the two Lac repressor binding heads as shown in fig. 5. We define the state variables 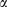 and 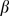 that describe the orientation of 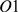 and 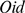, respectively, and that can adopt a value of either 1 or 2. The average 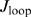 is then
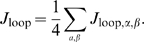
(3)An alternative to this scheme is to construct the ratio 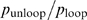. In the limit where the strongest operator, 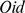, is always occupied, this ratio takes the simple, linear form
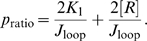
(4)This permits the determination of the 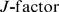 as the slope of a linear fit of the form without necessarily a need to obtain 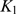. Below we discuss the validity of this particular model. For the remaining data points at loop lengths 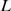 other than 306 bp, where no titration was done, we can use the relation
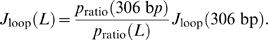
(5)Just like in the titration case, this relation allows to obtain 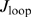 without knowing 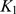, as long as we know at least one value of 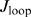 and its corresponding 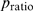.

The data shown in fig. 4 can be fit in several different ways as suggested by the three different formulae characterizing the looping probability given above. The fit shown in fig. 4 is a full nonlinear fit in which the parameters 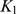, 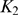 and 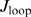 are treated as fitting parameters. Alternatively, using this same data of fig. 4, we can actually obtain the looping free energy, as well as the binding energies by fitting the data to eqn. 1. Note that these two descriptions are equivalent and each depends upon three unknown parameters. Once one set of parameters is known, in principle, the complementary parameters are also known. We find it convenient to work in terms of both languages because in some discussions it is useful to talk in terms of looping free energies, and in other contexts, in terms of the looping J-factor. Finally, we can fit the data corresponding to LacI concentrations of 10 pM and higher using the linear model from eqn. 4. The results of these different fits are shown in Table 1. These results are usefully contrasted with results of other experiments on the *lac* operon, which are also summarized in Table 1. We see from the table that the nonlinear model fails to constrain the value of 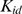 reliably. In the case of the 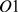 binding constants we see a difference of almost two orders of magnitude with published dissociation constants, which translates into a difference of roughly 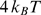 in the binding energy.

**Table 1:** Results from the LacI titration experiments.

Parameter	Nonlinear fit	Linear fit	Literature value
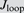	8.6±6.3 nM	52±40 nM	See fig. 12
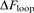	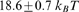	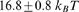	N/A
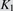	0.49±0.45 nM	3.0±2.5 nM	10∼22 pM [30], [57]–[62]
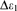	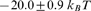	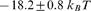	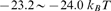
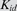	0.2±2.3 pM	N/A	2.4∼8.3 pM [92]
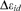	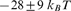	N/A	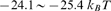

The probability of looping as a function of Lac repressor concentration shown in fig. 4 was fitted to the two non-linear models from eqns. 1 and 2. Both models were fit independently as a ways to check the robustness of the least-squares methods with respect to data reparametrization. A subset of the data corresponding to concentrations of LacI 10 pM and higher is fitted to the linear model shown in eqn. 4 and its statistical mechanics counterpart. See section S4 in the Supporting Information S1 for a discussion of the different data fitting approaches. The literature values of the dissociation constants for 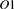 and 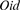 correspond to bulk binding assays performed in concentration ranges close to our TPM buffer conditions. The corresponding values for the binding energies of these operators are obtained from the dissociation constants using eqns. S5 and S11.

One of the challenges of single-molecule experiments like those described here is that the concentration of protein introduced into the system may not correspond to the actual concentration “seen” by the DNA that is tethered to the surface. For example, some of the protein might be lost as a result of nonspecific binding to the microscope coverslip. From the linear model shown in eqn. 4 it follows that any error in the concentration will translate linearly into an error in 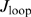 and 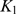. Therefore, in order for the above discrepancy to be explained solely by surface effects on the LacI concentration we would have to have a difference of between one and two orders of magnitude between the concentration of the stock that flowed into the chamber and the actual free concentration within the chamber.

**Figure 6:**
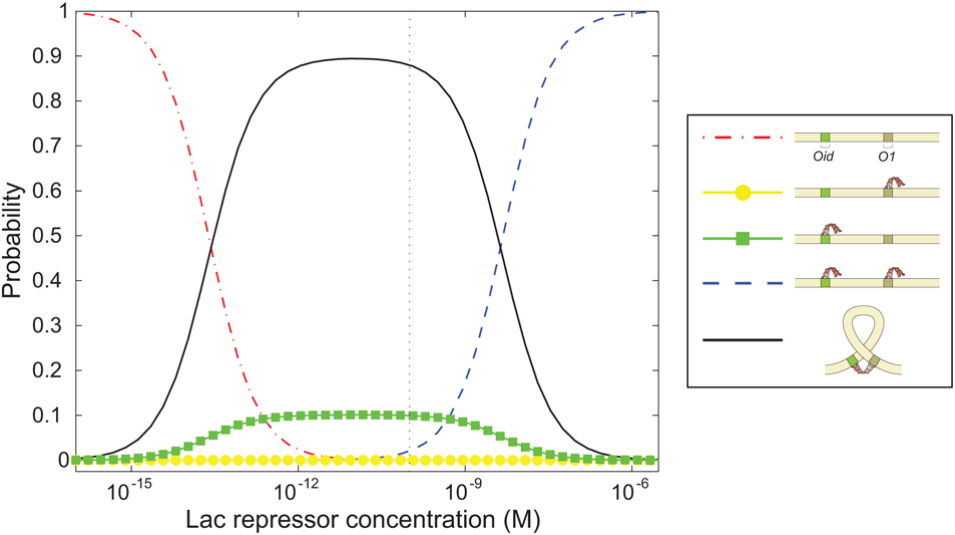
Probabilities for different states of Lac repressor and operator DNA. The curves show the probabilities of the five classes of microscopic states used in the statistical mechanics model based upon parameters shown in table 1. The vertical line corresponds to the concentration at which the loop length experiments in the remainder of the paper are performed.

Once the parameters that characterize the model are in hand, we can plot the probability of all five possible states as a function of the Lac repressor concentration as shown in fig. 6. This figure reveals that at the concentrations we normally use (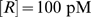), the system is dominated by the looped state and the state with single occupancy of 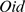. A detailed discussion of the significance of the looping free energies (or the 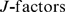) will follow later in the paper once we have explored the question of the length dependence of DNA looping in the *lac* operon.

### Length dependence

#### 1 bp resolution for a whole helical turn: *L*_loop_ = 300 bp to 310 bp

**Figure 7:**
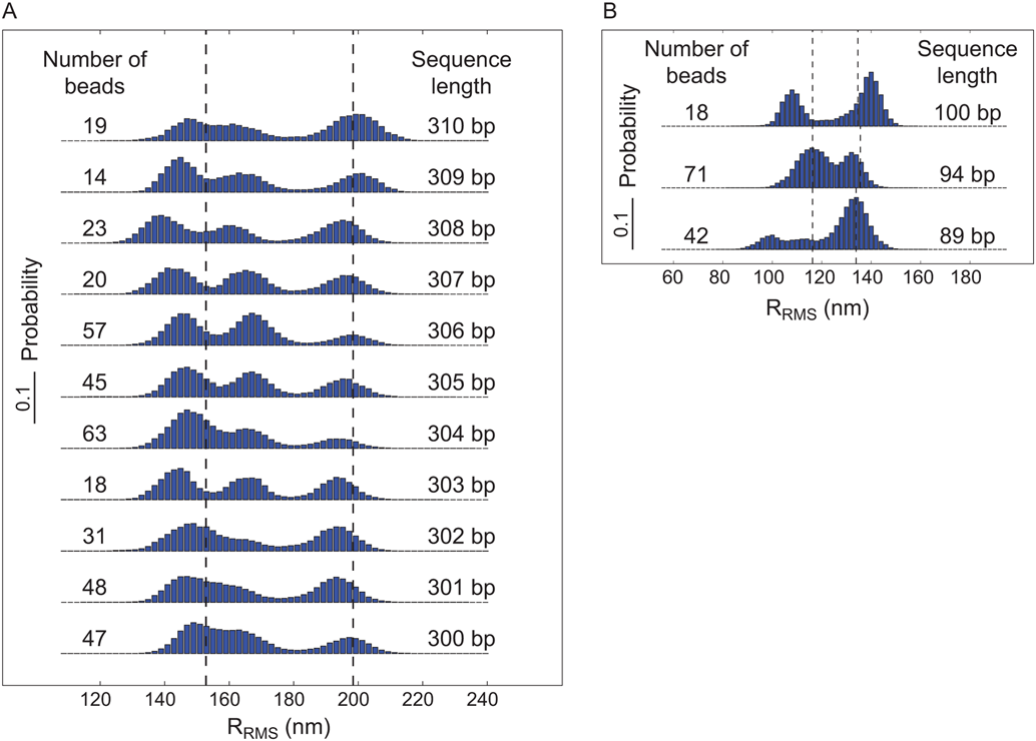
Length dependence of DNA looping. (A) Histogram of the tethered Brownian motion for DNAs with two Lac repressor binding sites spaced from 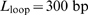 (bottom) to 310 bp (top). (B) Histogram of the Brownian motion for DNAs with two Lac repressor binding sites spaced at 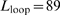, 94 and 100 bp. The two dashed lines represent the naively expected motion based on our calibration measurements for the full length tether and the same DNA when the center to center distance between operators is subtracted from the tether length. (Again see also fig. 11.) Representative traces for each of the lengths shown here can be found in the Supporting Information.

The beautiful *in vivo* repression experiments of [11] demonstrate that the length of the DNA loop formed by Lac repressor strongly affects the probability of loop formation (especially for loop lengths less than 150 bp). In particular, those authors (and others) [12], [13], [64], [65] have observed “phasing”: The relative orientations of the two operators changes the ease with which repressor can loop. Similar phasing effects have been observed in *in vitro* cyclization assays [3], [34], [66], [67]. What has not been clear is how to concretely and quantitatively relate these results on DNA mechanics from the *in vivo* and *in vitro* settings. Our idea was to systematically examine the same progression of DNA lengths that have been observed *in vivo*, but now using TPM experiments. To that end, we have measured TPM trajectories for a series of interoperator spacings measured in 1 bp increments. The results of this systematic series of measurements for DNAs harboring operators spaced over the range 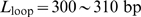 are shown in fig. 7 (as are the results for several shorter lengths to be discussed in the next section). Each plot shows the probability of the three states for a particular interoperator spacing.

**Figure 8:**
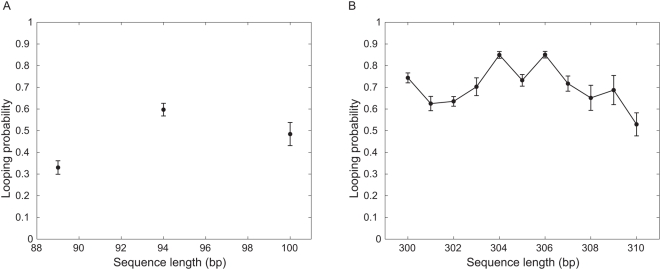
Looping probability 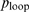, as a function of interoperator spacing. (A) Looping probability for short constructs. (B) Looping probability for one full helical repeat. These probabilities are obtained by averaging over the 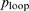 of each bead. The error bars correspond to the standard error associated with this magnitude. For more information see Supporting Information.

The data can be converted into a plot of the dependence of the looping probability on interoperator spacing as shown in fig. 8. This figure shows 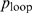 as a function of the DNA length between the two operators. The looping probability shows a weak dependence on the interoperator spacing but reveals no conclusive signature of phasing; to really detect such phasing with confidence, however, would require more measurements in single basepair increments. The maximum looping is achieved when the two binding sites are 306 bp apart, suggesting that at this distance, the two sites are in an optimal phasing orientation for binding of the two heads of Lac repressor. The ability to form stable out-of-phase (two binding sites are on the opposite side of the DNA) loops with only a small reduction in stability is consistent with previous studies [26]. The relatively stable looping over the entire helical repeat is also consistent with the relatively constant repression level *in vivo* for similar interoperator spacing [11].

**Figure 9:**
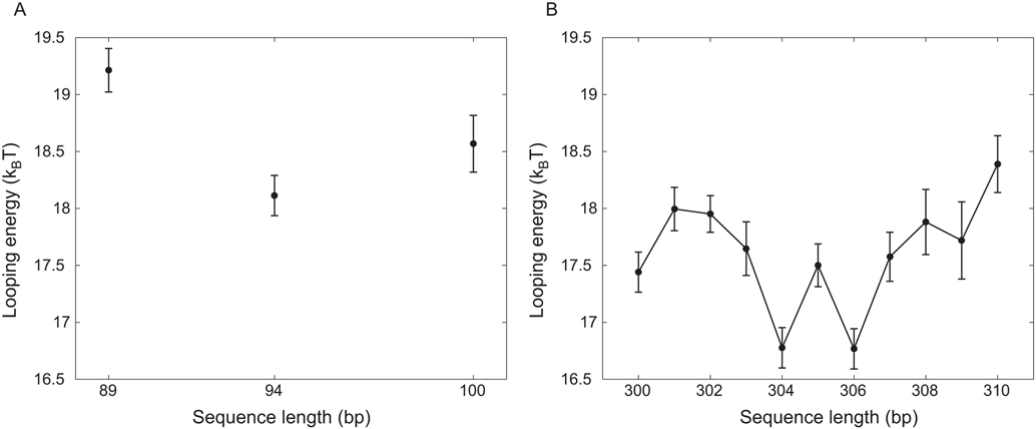
Length dependence of free energy of looping, defined via eqn. 1 with choice of reference concentration 1 M. (A) Looping free energy for short constructs. (B) Looping free energy for a full helical repeat.

**Figure 10:**
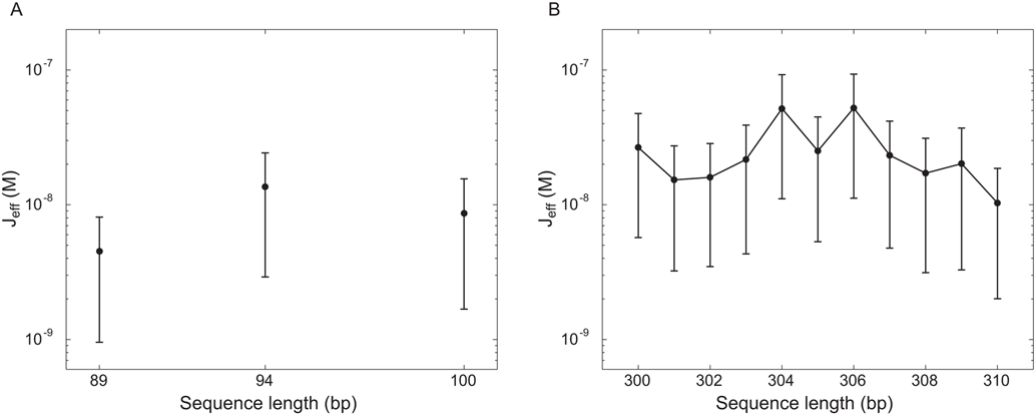
Looping *J*-factor resulting from TPM measurements. A) Effective 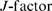 for looping resulting from TPM data on short constructs. (B) Effective 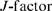 for looping resulting from TPM data on a full helical repeat.

As already indicated in Table 1, the looping probability can be converted into a corresponding looping free energy based on the statistical mechanics model described above and culminating in eqn. 1. The results of such calculation are shown in fig. 9. The measurements on length dependence permit us to go beyond the concentration dependence measurements by systematically exploring how the phasing of the two operators impacts the free energy of DNA looping. One might expect that when the two operators are on opposite sides of the DNA, additional twist deformation energy is required to bring the operators into good registry for Lac repressor binding. Our results show that the phasing effect imposes an energy penalty 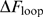 that differs by only about 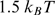 between the in-phase and out of phase cases. An alternative interpretation of these same results on looping probability is offered by the 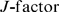 for looping as shown in fig. 10.

To get a feel for the energy scale associated with twist deformations, we perform a simple estimate. Twisting DNA for a torsional angle 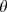 requires energy
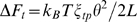
(6)where 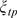 is the torsional persistence length for double stranded DNA, which is around 250 bp [68]–[70]. 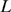 is the DNA length. For half a helical turn twist, 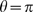 and 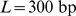. The energy introduced for half a helical turn is around 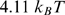. Our experimentally determined looping energy difference between in-phase and out-of-phase DNA, about 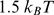, is indeed comparable in magnitude to this estimate. Our simple estimate is high, in part because it neglects the fact that in addition to twisting, a loop can writhe to accommodate a nonideal operator phasing. Additionally, the observed small magnitude of our observed phasing modulation may reflect partially canceling out-of-phase contributions of different topologies [39], not a low free energy cost for twisting. Finally, the Lac repressor itself is flexible, and so can partially compensate for nonideal phasing.

#### Sub-persistence length loops

One of the intriguing facts about the architecture of regulatory motifs that involve DNA looping is that often the loops formed in these systems have DNA lengths that are considerably shorter than the persistence length of DNA (i.e. 150 bp). For example, in the *lac* operon, one of the three wild-type loops has a length of 92 bp. However, this trend goes well beyond the *lac* operon as is seen for a variety of different architectures found in *E. coli*, for example [1]. As a result, it is of great interest to understand the interplay between transcriptional regulation and corresponding mechanical manipulations of DNA this implies.

So far, we have considered loops that are roughly two-fold larger than the persistence length through our investigation of one full helical repeat between 300 and 310 bp. To begin to develop intuition for the mechanism of loop formation in the extremely short loops exhibited in many regulatory architectures, we have examined three different lengths: 89, 94 and 100 bp. One of the reasons that the examination of these loops is especially important is that it has been speculated that the *in vivo* formation of these loops either requires special supercoiling of the DNA or the assistance of helper proteins that prebend the DNA [1]. However, as indicated by the TPM results shown in fig. 7(B), even in our controlled *in vitro* setting, where neither of these mechanisms can act, Lac repressor is nevertheless able to form DNA loops. The essence of these experiments is identical to those described earlier in the paper except that now the overall tether lengths are shorter so as to ensure that the loops are detectable. (Representative TPM trajectories for these lengths are shown in the Supporting Information S1.) It is clear from the histograms that of the three lengths we have investigated, loop formation is most favorable at 94 bp. Interestingly, it also appears that different loops are being formed for the in-phase and out-of-phase cases as evidenced by the changes of relative strengths among the looping peaks for the different constructs. The looping free energy and 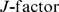 for looping for these short constructs are shown in figs. 9(A) and 10(A).

### Analysis of the TPM Experiment

Both the observed length and sequence dependence of the formation of a repression complex are intriguing from the perspective of DNA mechanics. In particular, DNA is not a passive mechanical bystander in the process of transcriptional regulation. To better understand the experiments carried out here and how they might shed light on the interplay of transcription factors and their target DNA, we have appealed to two classes of models: i) statistical mechanics models of the probability of DNA-repressor complex formation which depends upon the looping free energy (these models were invoked earlier in the paper to determine the looping free energy) and ii) Monte Carlo simulations of the TPM experiment itself which include the energetics of the bent DNA and excluded volume interactions of the bead with the coverslip. Our Monte Carlo calculations allow us to compute how easily loops form, based on a mathematical model of DNA elasticity. For illustration, we have chosen a linear-elasticity model, that is, a model in the class containing the wormlike chain, but any other elastic theory of interest can be used with the same calculation strategy. Details of these calculations appear in [39].

One of the puzzles that has so far been unresolved concerning DNA mechanics at short scales is whether *in vivo* and *in vitro* experiments tell a different story. In particular, *in vivo* experiments, in which repression of a given gene is measured as a function of the interoperator spacing [11], [12], have the provocative feature that the maximum in repression (or equivalently the minimum in looping free energy) correspond to interoperator spacings that are shorter than the persistence length. Some speculate that this *in vivo* behavior results from the binding of helper proteins such as the architectural proteins HU, H-NS or IHF [1], [12], [13] or the control of DNA topology through the accumulation of twist. In the TPM measurements reported here, there are neither architectural proteins nor proteins that control the twist of the DNA. As a result, these experimental results serve as a jumping off point for a quantitative investigation of whether DNA at length scales shorter than the persistence length behaves more flexibly than expected on the basis of the wormlike chain model. To address this question, we performed a series of simulations of the probability of DNA looping for short, tethered DNAs like those described here using, a variant of the wormlike chain model to investigate the looping probability. Our theoretical model used *no fitting parameters*; the few parameters defining the model were obtained from other, non-TPM, experiments.

The fraction of time spent in the looped configuration is controlled by several competing effects. For example, suppose that a repressor tetramer is bound to the stronger operator, 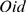. Shortening the interoperator spacing reduces the volume over which the other operator (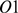) wanders relative to the second binding site on the repressor, increases the apparent local “concentration” of free operator in the neighborhood of that binding site, and hence enhances looping. But decreasing the interoperator spacing also has the opposite effect of discouraging looping, due to the larger elastic energy cost of forming a shorter loop. Moreover, a shorter overall DNA construct increases the entropic force exerted by bead–wall avoidance, again discouraging looping [71]. To see what our measurement of this looping equilibrium tells us, we therefore needed to calculate in some detail the expected local concentration of operator (the “looping 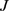 factor”) based on a particular mathematical model of DNA elasticity. Traditionally, DNA has been modeled mathematically as a thin, elastic solid body with a classical Hooke-law elastic energy function. Because classical elasticity theory assumes that energy is a quadratic (“harmonic”) function of strain, such models are collectively called “harmonic-elasticity” models; one example is the wormlike chain model. Accordingly, we used a harmonic-elasticity model, to see if it could adequately explain our results, or if, on the contrary, some non-harmonic model (for example the one proposed in [72], [73]) might be indicated.

To perform the required calculation, we modified the Gaussian sampling method previously used in [71], [74]–[76] (see section S6 and [39]). Our code generated many simulated DNA chains, applied steric constraints [71], and reported what fraction of accepted chain/bead configurations had the two operator sites at the correct relative position and orientation for binding to the tetramer, which was assumed to be rigidly fixed in the form seen in PDB structure 1LBG [77]. Once this fraction has been computed, it is straightforward to relate it to the looping 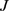 factor [39]. Note that the beauty of the looping 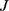 factor is that it is independent of the particular binding strengths of the different operators. To generate the simulated chains, we assumed a linear (harmonic, or wormlike-chain type) elastic energy function at the junctions in a chain of finite elements. Our energy function accounted for the bend anisotropy and bend–roll coupling of DNA, and yielded a value for the persistence length ξ = 44 nm appropriate for our experiment's buffer conditions [78], [79]. Our model did not account for sequence dependence. We assume that this simplification is appropriate for comparison to our experimental results for the case of the 300 bp constructs and the 90 bp constructs with the sequence E8, but not with the sequence TA. The simulation treated the bead and the microscope slide as hard walls and accounted for bead–wall, bead–chain, and wall–chain avoidance; we did not consider any interactions involving the repressor tetramer other than binding.

**Figure 11:**
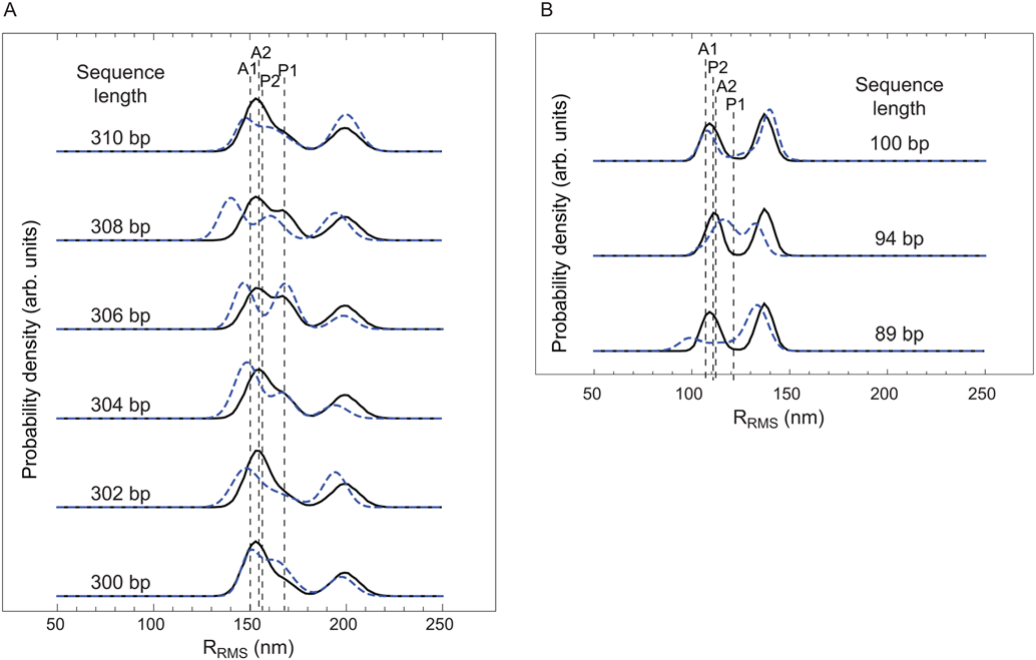
Theory and experiment for the probability density functions of RMS bead excursion for (A) our six “long chain” constructs and (B) our three “short chain” constructs. *Blue dashed curves* show the data in fig. 7, represented as sums of three Gaussians. *Black curves* show our theoretically predicted distributions. Because our simulation results were not fits to the data, they did not reproduce perfectly the ratio of looped to unlooped occupancies. For visualization, therefore, we have adjusted this overall ratio by a factor common to all six curves. This rescaling does not affect the locations of the peaks, the relative weights of the two looped-state peaks, nor the dependences of weights on loop length 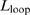, all of which are zero-fit-parameter predictions of our model. The model yields these histograms as the sum of five contributions, corresponding to the four looped topologies and the unlooped state. The topologies correspond to the different geometries shown in fig. 5. The separate RMS displacements for each individual loop topology for the 89 bp case in (A) and for the 300 bp case in (B) are also shown, labeled according to the scheme in [36].

The symmetry of each LacI dimer implies four energetically equivalent ways for the two operators to bind when forming a loop, and hence four topologically distinct loop configuration classes [35]–[39]. We first asked whether this multiplicity of looped states could explain the general structure of the excursion distributions seen in fig. 7. Accordingly, we made histograms of the distance between wall attachment point and bead center for our simulated chains. Fig. 11 shows a subset of the same experimental data seen in fig. 7, together with the simulation results. Although the correspondence is not perfect, it is clear that the simple physical model of looping outlined above can account for many features of the data, for example the locations of the looped peaks and their relative strengths, including the variation as loop length is changed. We acknowledge that we have no definitive reply to the argument that the apparent direct transitions between the B and M peaks of our distributions seem to require an open-to-closed conformational switch in the tetramer [26]. We merely point out that the existence of three peaks in the distribution, with the the observed locations, is not by itself conclusive evidence of such a switch. (Indeed, Villa *et al.* have argued that the opening transition does not occur [80].)

**Figure 12:**
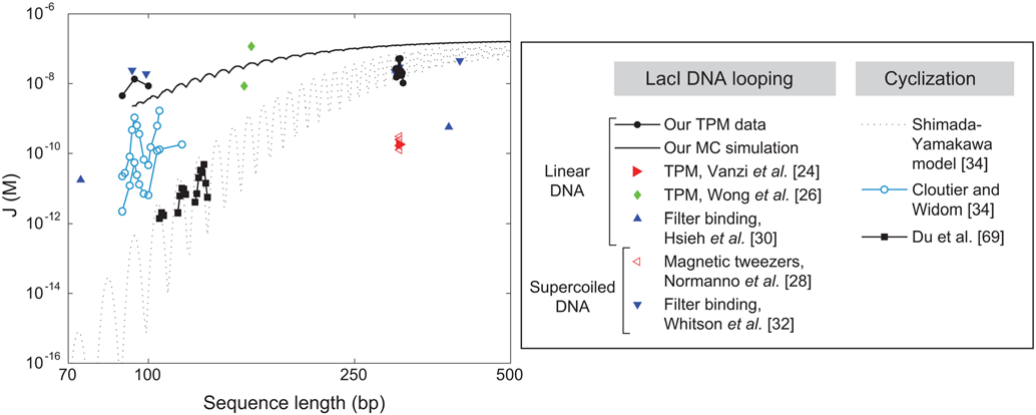
Effective *J*-factor from different experiments. Although the 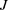 factor obtained from cyclization experiments is not directly comparable to the looping 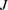 factor studied in this paper (due to the differences in geometry), we present the two quantities together as functions of loop length to summarize the work from many groups. Error bars have been omitted for clarity. The filter binding data is an order of magnitude estimate.

We were also interested to see if the high incidence of looping observed in our experiments on short (sub persistence length) loops was compatible with the hypotheses of harmonic DNA elasticity and fixed repressor geometry, or if on the contrary it demanded some modification to those hypotheses. Accordingly, we asked the simulation to compute the average 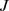 factor for loop lengths near 305 bp, and also for loop lengths near 95 bp. As discussed in ref. [39], the result of the simulation was that the ratio of these quantities is 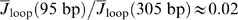. In contrast, fig. 10 shows that the experimental ratio is ≈0.35±0.1, roughly 20-fold larger than the theoretical value. Our experimental results and those interpolated from our MC calculations for 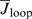 as a function of loop length are shown in fig. 12.

We conclude that the hypotheses of linear elasticity, a rigid protein coupler, and no nonspecific DNA–repressor interactions, cannot explain the high looping incidence seen in our experiments. (Special DNA sequences loop even more easily than the random sequences reported here.) One possible explanation, for which other support has been growing, is the hypothesis of DNA elastic breakdown at high curvature [72], [73], [81]. An alternative hypothesis is that for our shorter loops, *both* the lower and the intermediate peaks in our distributions of bead excursion correspond to the some alternative, “open” conformation of the repressor tetramer [35], [36], [45], [82]–[85]. To be successful, however, this hypothesis would have to pass the same quantitative hurdles to which we subjected our hypotheses.

## Discussion

The regulatory regions on DNA can often be as large as (or even larger than) the genes they control. The relation between the biological mechanisms of transcriptional control and the physical constraints put on these mechanisms as a result of the mechanical properties of the DNA remains unclear. One avenue for clarifying action at a distance by transcription factors is systematic single-molecule experiments, which probe the dynamics of loop formation for different DNA architectures (i.e. different sequences, different transcription factor binding strengths, different distances between transcription factor binding sites) to complement systematic *in vivo* experiments that explore these same parameters. In this paper, we have described an example of such a systematic series of measurements, which begins to examine how the formation of transcription factor-DNA complexes depend upon parameters such as transcription factor concentration and the length of the DNA implicated in the complex.

In the case of the *lac* operon, our *in vitro* measurements demonstrate that the formation of the looped repressor-DNA complex does not require any helper proteins, nor does it call for supercoiling of the DNA (as appears to be required in other bacterial regulatory architectures [5], [6]). Further, we find that even in the absence of these mechanisms, which can only enhance the probability of loop formation, the formation of DNA loops by Lac repressor occurs more easily than would be expected on the basis of traditional views of DNA elasticity. A summary of the various measurements of short-length DNA cyclization and looping is shown in fig. 12. The idea of this figure is to present the diversity of data that weighs in on the subject of short length DNA elasticity. In particular, several sets of controversial measurements on DNA cyclization present different conclusions on the ease of this process at lengths of roughly 100 bp. Note that in addition, we have included both the theoretical cyclization J-factor and looping J-factor. The looping J-factor reveals that because of the less restrictive looping geometry (end points are not at same point in space and the tangents are not constrained to be equal), looping costs less free energy than does cyclization. TPM experiments like those presented here offer another avenue to resolve this issue, one that does not involve the complex ligase enzyme, the need to ensure a specific kinetic regime, nor other subtleties of the ligation reaction inherent in cyclization measurements. However, as seen in the figure, even here there are unexplained discrepancies between different TPM experiments which call for continued investigation. One observation from our own work that could have an important bearing on the differences in TPM results between different groups is that there is a substantial temperature dependence to the looping probabilities and different groups may be working at different temperatures.

Several intriguing mysteries remain which demand both further experimentation as well as theoretical analysis, e.g.: i) why are the probabilities of DNA loop formation systematically higher than would be expected on the basis of traditional arguments about DNA elasticity, and ii) what is the significance of three repressor binding sites in the wild-type *lac* operon? To explore these questions, TPM experiments with different DNA sequences between the two operators, as well as with Lac repressor mutants that are less flexible, would go a long way towards clarifying the mechanisms at work and would provide a basis for examining the even richer action at a distance revealed in the eukaryotic setting.

## Materials and Methods

### Plasmid DNAs

**Figure 13:**
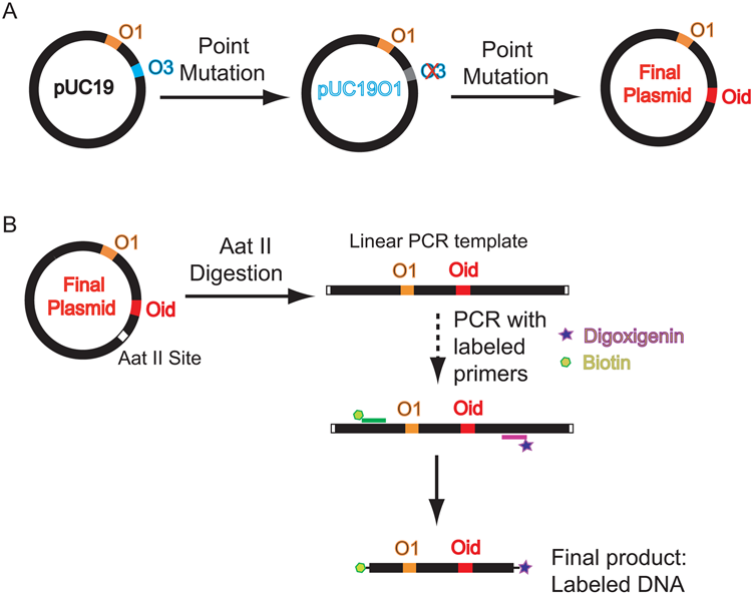
Synthesis of DNA construct. A) Schematic of the procedure for construction of the plasmid with two Lac repressor binding sites. (B) Schematic of the protocol for producing labeled DNA using a PCR reaction with labeled primers.

Plasmid DNAs, bearing two Lac repressor binding sites spaced at a designed distance, are created using a point mutation method (QuikChange site-directed mutagenesis, Stratagene) on plasmid pUC19. Plasmid pUC19 was chosen as a starting template because it is not only a high copy plasmid but also contains two Lac repressor binding sites: 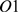 and 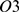. The procedure for creating two binding sites separated by the desired distance from template pUC19 is illustrated in fig. 13(A). We first mutate six basepairs in the 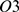 site converting it to 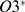 in a way that eliminates the binding affinity for this site [86]. The resulting plasmid is called pUC19O1 indicating it only has a single 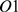 site. To construct another binding site on the pUC19O1 plasmid, we replace 20 bp with the Lac repressor binding sequence 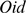 at a series of locations differing by 1 bp increments in their distance from 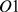 using the mutagenesis method again. For some of the secondary site construction, we have to use either deletion or addition from already made plasmids with two designed binding sites. The details on primers and templates used in this process are listed in Table 2. The final product contains two binding sites 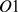 and 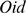 spaced at the desired distance.

**Table 2:** Materials used in the mutagenesis process for creating plasmids with two Lac repressor binding sites.

Molecule	Primer	Template	Action	Resulting Molecule
pUC19O1	Mut0	pUC19	Replace	O1
pUC300	Mut1	pUC301	Delete 1 bp	O1-300bp-Oid
pUC301	Mut2	pUC19O1	Replace	O1-301bp-Oid
pUC302	Mut3	pUC19O1	Replace	O1-302bp-Oid
pUC303	Mut4	pUC19O1	Replace	O1-303bp-Oid
pUC304	Mut5	pUC19O1	Replace	O1-304bp-Oid
pUC305	Mut6	pUC19O1	Replace	O1-305bp-Oid
pUC306	Mut7	pUC19O1	Replace	O1-306bp-Oid
pUC307	Mut8	pUC19O1	Replace	O1-307bp-Oid
pUC308	Mut9	pUC19O1	Replace	O1-308bp-Oid
pUC309	Mut10	pUC308	Add 1bp	O1-309bp-Oid
pUC310	Mut11	pUC308	Add 2 bp	O1-310bp-Oid

Primer sequences(5′→3′):

Mut0: ctaactcacattaattgcgttgAgctcGAGgTTcgctttccagtc.

Mut1: catacgagccggaa (G) cataaagtgtaaagc.

Mut2: ctcggaaagaaca AATTGTGAGCGCTCACAATT aaggccaggaacc.

Mut3: ctcggaaagaacat AATTGTGAGCGCTCACAATT aggccaggaaccg.

Mut4: cggaaagaacatg AATTGTGAGCGCTCACAATT ggccaggaaccgt.

Mut5: ggaaagaacatgt AATTGTGAGCGCTCACAATT gccaggaaccgta.

Mut6: gaaagaacatgtg AATTGTGAGCGCTCACAATT ccaggaaccgtaa.

Mut7: cggaaagaacatgtga AATTGTGAGCGCTCACAATT caggaaccgtaaaaag.

Mut8: ggaaagaacatgtgag AATTGTGAGCGCTCACAATT aggaaccgtaaaaagg.

Mut9: gaaagaacatgtgagc AATTGTGAGCGCTCACAATT ggaaccgtaaaaaggc.

Mut10: catacgagccggaag [C] cataaagtgtaaagc.

Mut11: catacgagccggaag [CG] cataaagtgtaaagc.

The capital letters in the primer sequences indicate the mutations. ‘()’ indicates bp deletion and ‘[ ]’ indicates bp addition. The inter-operator distance indicated here is the distance between two inner edges of the operators instead of center to center distance that is commonly used in *in vivo* experiments [11]–[13], [86], [93].

The short loop DNA (89, 94 and 100 bp) was constructed in the following way. Plasmid pZS22-YFP was kindly provided by Michael Elowitz. The main features of the pZ plasmids are located between unique restriction sites [87]. The YFP gene comes from plasmid pDH5 (University of Washington Yeast Resource Center [88]).

**Figure 14:**
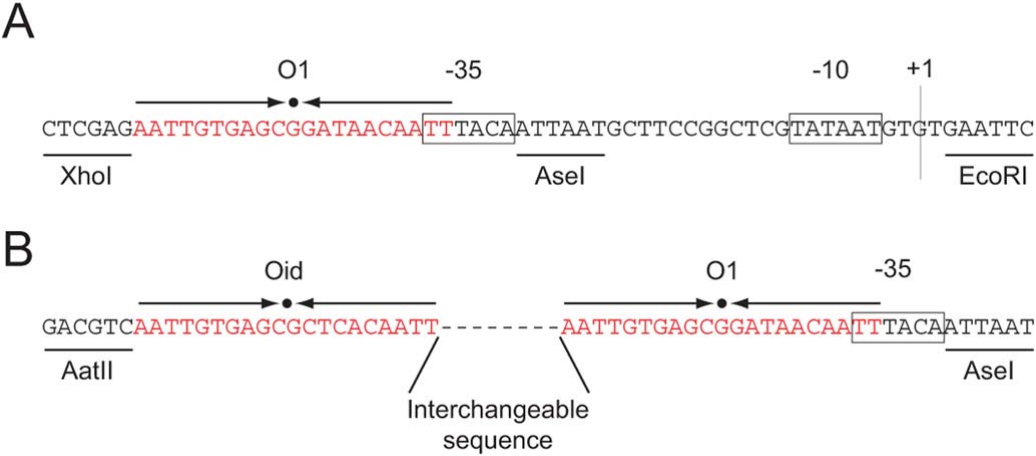
Promoter regions of the different short loop constructs. (A) Promoter region of pZS25-YFP which has a variant of the lacUV5 promoter and an 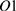 operator upstream overlapping the −35 region. (B) Final construct that allows to insert arbitrary DNA sequences between a 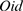 and 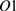 operators.

A variant of the lacUV5 promoter [10] was synthesized and placed between the EcoRI and XhoI sites of pZS22-YFP in order to create pZS25'-YFP. This promoter included the −35 and −10 regions of the lacUV5 promoter, an AseI site between the two signals and a 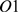 operator at position −45 from the transcription start as shown in fig. 14(A).

The random sequence E8-89 [34] was obtained by PCR from a plasmid kindly provided by Jonathan Widom. The primers used had a flanking AatII site and 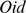 operator upstream and a flanking 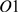 operator, −35 region and AseI site downstream. This PCR product was combined with the appropriate digest of pZS25'-YFP to give raise to 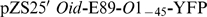. This is shown schematically in fig. 14(B). Finally, the different lengths used by Cloutier and Widom [3], [34] were generated from this template using site directed mutagenesis.

### Construction of labeled DNAs

**Figure 15:**
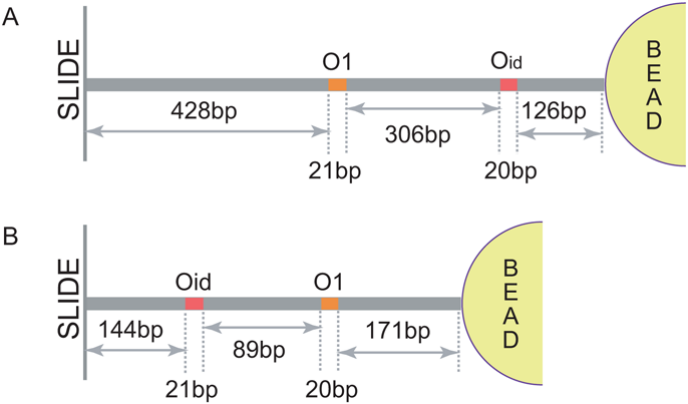
Examples of the tether constructs used. (A) In the long distance constructs 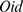 was displaced keeping the total construct length constant. (B) In the short distance constructs the sequence between the operators was altered, which results in each construct having a slightly different total length. (Drawings not to scale.

In TPM experiments, DNA is linked between the substrate and a bead. Two pairs of linkers: biotin-streptavidin and digoxigenin-anti-digoxigenin, are chosen to permit specific linkage of the DNA to a polystrene microsphere and glass coverslip, respectively. As illustrated in fig. 13(B), PCR was used to amplify such labeled DNA with two modified primers. Each primer is designed to be about 20 bp in length and linked with either biotin or digoxigenin at the 5′ end (Eurofins MWG Operon). In the case of the long sequence constructs, in order to optimize the PCR reaction linearized plasmids with an AatII cut are used as the template. Detailed information concerning the design of our PCR reactions is listed in Table 3 and the constructs are shown schematically in fig. 15. The PCR products were then purified by gel extraction (QIAquick Gel Extraction Kit, QIAGEN) and the concentration of the DNA was measured using quantitative DNA electrophoresis.

**Table 3:** Materials used in amplifying labeled DNA using PCR.

Molecule	Template	Length(bp)	Resulting
pUC300L1	pUC300	900	Dig - 427bp-O1-300bp-Oid-132bp - Bio
pUC301L1	pUC301	901	Dig - 427bp-O1-301bp-Oid-132bp - Bio
pUC302L1	pUC302	901	Dig - 427bp-O1-302bp-Oid-131bp - Bio
pUC303L1	pUC303	901	Dig - 427bp-O1-303bp-Oid-130bp - Bio
pUC304L1	pUC304	901	Dig - 427bp-O1-304bp-Oid-129bp - Bio
pUC305L1	pUC305	901	Dig - 427bp-O1-305bp-Oid-128bp - Bio
pUC306L1	pUC306	901	Dig - 427bp-O1-306bp-Oid-127bp - Bio
pUC307L1	pUC307	901	Dig - 427bp-O1-307bp-Oid-126bp - Bio
pUC308L1	pUC308	901	Dig - 427bp-O1-308bp-Oid-125bp - Bio
pUC309L1	pUC309	902	Dig - 427bp-O1-309bp-Oid-125bp - Bio
pUC310L1	pUC310	903	Dig - 427bp-O1-310bp-Oid-125bp - Bio
E8-89	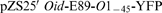	445	Dig - 144bp-Oid-89bp-O1-171bp - Bio
E8-94	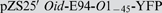	450	Dig - 144bp-Oid-94bp-O1-171bp - Bio
E8-100	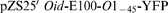	456	Dig - 144bp-Oid-100bp-O1-171bp - Bio

Primer sequences(5′→3′):

Plen901F: Dig - ACAGCTTGTCTGTAAGCGGATG.

Plen901R: Bio - CGCCTGGTATCTTTATAGTCCTGTC.

PF1: Dig - ATGCGAAACGATCCTCATCC.

PR1: Bio - GCATCACCTTCACCCTCTCC.

The inter-operator distances indicated here is the distance between two inner sides of the operators instead of center to center distance. Primers Plen901F and Plen901R were used for the long distance constructs. Primers PF1 and PR1 were used for the short distance constructs.

### TPM sample preparation

TPM sample preparation involves assembly of the relevant DNA tethers and their associated reporter beads. Streptavidin coated microspheres (Bangs lab) of diameter 490 nm served as our tethered particle. Prior to each usage, a buffer exchange on the beads was performed by three cycles of centrifugation and resuspension in TPB buffer (20 mM Tris-acetate, pH = 8.0, 130 mM KCl, 4 mM MgCl_2_, 0.1 mM DTT, 0.1 mM EDTA, 20 µg/ml acetylated BSA (Sigma-Aldrich), 80 µg/ml heparin(Sigma-Aldrich) and 0.3% biotin-free casein. Biotin-free casein colloidal buffer (5% casein colloid with 0.001% Merthiolate, RDI, Flanders, NJ) was used as a cassein source. This combination of reagents was chosen in an attempt to maximize sample yield and longevity, while minimizing non-specific adsorption of DNA and microspheres onto the coverslip.

**Figure 16:**
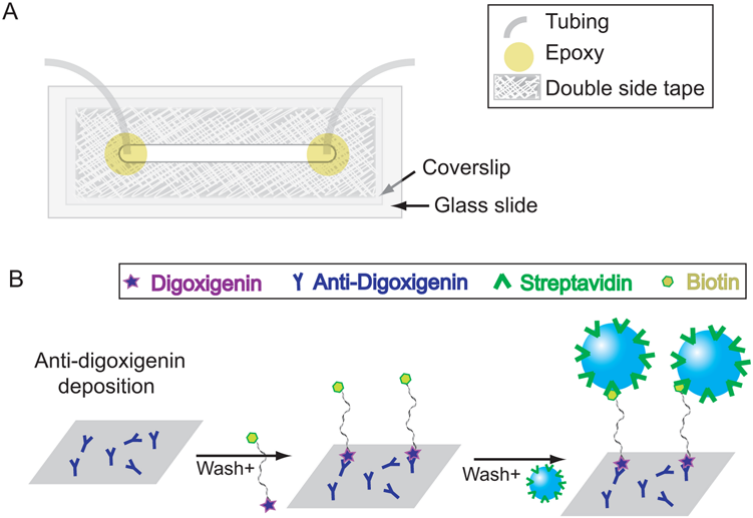
Illustration of TPM sample preparation. (A) Sketch of the flow cell. (B) The scheme for making DNA tethers.

Tethered particle samples were created inside a 20–40 µl flow cell made out of a glass slide with one hole near each end, glass coverslip, double-sided tape and tygon tubing. The coverslip and glass slide were cleaned with plasma cleaning for 4 minutes and then the flow cell was constructed as shown in fig. 16(A). Two tygon tubes serving as an input and output were inserted into the holes on the glass slide and sealed with epoxy. A reaction chamber was created by cutting a channel on the double sided tape, which glues the coverslip and glass slide together. Making the end of the channel round and as close to the holes of the glass slide as possible is important to avoid generating bubbles. The flow cell was then heated for about 20 seconds to seal securely.

For DNA tether assembly, the flow chamber was first incubated with 20 µg/mg polyclonal anti-digoxigenin (Roche) in PBS buffer for about 25 minutes, and then rinsed with 400 µl wash buffer (TPB buffer with no casein) followed by 400 µl of TPB buffer. 250 µl of labeled DNA in TPB buffer with about 2 pM concentration was flushed into the chamber and incubated for around 1 hour. After washing with 750 µL TPB buffer to remove any unbound DNA, a 10 pM solution of beads were introduced into the chamber and incubated for 20 minutes. Finally, unbound microspheres were removed by flushing the chamber with 1 mL TPB buffer. For looping experiments, 0.5 mL∼1 mL LRB buffer (10 mM Tris-Hcl, pH 7.4, 200 mM KCl, 0.1 mM EDTA, 0.2 mM DTT, 5% DMSO and 0.1% biotin-free casein) containing the desired concentration of Lac repressor (a kind gift from Kathleen Matthews' lab) was then flushed into the chamber and incubated about 15 minutes before observation. Although we were able to measure the overall concentration of Lac repressor used in the experiments, the more important quantity is the concentration of active repressor which we were unable to successfully measure other than through the looping assay itself. Each flow cell preparation would typically allow to acquire data on ten tethers.

### Data Acquisition and Processing

The motion of the bead is recorded through a Differential Interference Contrast (DIC) microscope at 30 frames per second. The position of the bead is tracked in the x−y plane using a cross-correlation method [89] and recorded as raw data for further analysis. Such raw positional data are subject to a slow drift due to vibrations of the experimental apparatus. A drift correction is then applied using a high pass first-order Butterworth filter at cutoff frequency 0.1 Hz [24]. From the filtered data, 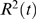 is then calculated as 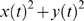 and a running average 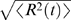 is obtained using a Gaussian filter at cutoff frequency 0.033 Hz [24], [90], which corresponds to the standard deviations of the filter's impulse response time of 4 s. The traces shown in this paper are all obtained in this way.

## Supporting Information

Supporting Information S1 Supporting Information S1
(10.68 MB PDF)

Movie S1 (3.61 MB MPG)

## References

[1] Garcia HG, Grayson P, Han L, Inamdar M, Kondev J (2007). Biological consequences of tightly bent DNA: The other life of a macromolecular celebrity.. Biopolymers.

[2] Segal E, Fondufe-Mittendorf Y, Chen L, Thastrom A, Field Y (2006). A genomic code for nucleosome positioning.. Nature.

[3] Cloutier TE, Widom J (2004). Spontaneous sharp bending of double-stranded DNA.. Mol Cell.

[4] Adhya S (1989). Multipartite genetic control elements: communication by DNA loop.. Annu Rev Genet.

[5] Schleif R (1992). DNA looping.. Annu Rev Biochem.

[6] Matthews KS (1992). DNA looping.. Microbiol Rev.

[7] Zeller RW, Griffith JD, Moore JG, Kirchhamer CV, Britten RJ (1995). A multimerizing transcription factor of sea urchin embryos capable of looping DNA.. Proc Natl Acad Sci U S A.

[8] Dunn TM, Hahn S, Ogden S, Schleif RF (1984). An operator at −280 base pairs that is required for repression of araBAD operon promoter: addition of DNA helical turns between the operator and promoter cyclically hinders repression.. Proc Natl Acad Sci USA.

[9] Ptashne M (2004). A genetic switch: phage lambda revisited.

[10] Müller-Hill B (1996). The *lac* Operon: a short history of a genetic paradigm.

[11] Müller J, Oehler S, Müller-Hill B (1996). Repression of *lac* promoter as a function of distance, phase and quality of an auxiliary *lac* operator.. J Mol Biol.

[12] Becker NA, Kahn JD, Maher LJ (2005). Bacterial repression loops require enhanced DNA exibility.. J Mol Biol.

[13] Becker NA, Kahn JD, Maher r LJ (2007). Effects of nucleoid proteins on DNA repression loop formation in *escherichia coli*.. Nucleic Acids Res.

[14] Schafer DA, Gelles J, Sheetz MP, Landick R (1991). Transcription by single molecules of RNA polymerase observed by light microscopy.. Nature.

[15] Yin H, Landick R, Gelles J (1994). Tethered particle motion method for studying transcript elongation by a single RNA polymerase molecule.. Biophys J.

[16] Vanzi F, Vladimirov S, Knudsen CR, Goldman YE, Cooperman BS (2003). Protein synthesis by single ribosomes.. RNA.

[17] Pouget N, Dennis C, Turlan C, Grigoriev M, Chandler M (2004). Single-particle tracking for DNA tether length monitoring.. Nucleic Acids Res.

[18] Blumberg S, Tkachenko AV, Meiners JC (2005). Disruption of protein-mediated DNA looping by tension in the substrate DNA.. Biophys J.

[19] Pouget N, Turlan C, Destainville N, Salome L, Chandler M (2006). Is911 transpososome assembly as analysed by tethered particle motion.. Nucleic Acids Res.

[20] van den Broek B, Vanzi F, Normanno D, Pavone FS, Wuite GJ (2006). Real-time observation of DNA looping dynamics of Type IIE restriction enzymes NaeI and NarI.. Nucleic acids research.

[21] Tolic-Norrelykke SF, Rasmussen MB, Pavone FS, Berg-Sorensen K, Oddershede LB (2006). Stepwise bending of DNA by a single TATA-box binding protein.. Biophys J.

[22] Guerra RF, Imperadori L, Mantovani R, Dunlap DD, Finzi L (2007). DNA compaction by the nuclear factor-Y.. Biophys J.

[23] Finzi L, Gelles J (1995). Measurement of lactose repressor-mediated loop formation and breakdown in single DNA molecules.. Science.

[24] Vanzi F, Broggio C, Sacconi L, Pavone FS (2006). Lac repressor hinge exibility and DNA looping: single molecule kinetics by tethered particle motion.. Nucleic Acids Res.

[25] Zurla C, Franzini A, Galli G, Dunlap DD, Lewis DEA (2006). Novel tethered particle motion analysis of cI protein-mediated DNA looping in the regulation of bacteriophage lambda.. Journal of Physics-Condensed Matter.

[26] Wong OK, Guthold M, Erie DA, Gelles J (2008). Interconvertible lac repressor-DNA loops revealed by single-molecule experiments.. PLoS Biol.

[27] Zurla C, Samuely T, Bertoni G, Valle F, Dietler G (2007). Integration host factor alters LacI-induced DNA looping.. Biophys Chem.

[28] Normanno D, Vanzi F, Pavone FS (2008). Single-molecule manipulation reveals supercoilingdependent modulation of *lac* repressor-mediated DNA looping.. Nucleic Acids Res.

[29] Krämer H, Niemöller M, Amouyal M, Revet B, von Wilcken-Bergmann B (1987). *Lac* repressor forms loops with linear DNA carrying two suitably spaced *lac* operators.. EMBO J.

[30] Hsieh WT, Whitson PA, Matthews KS, Wells RD (1987). Inuence of sequence and distance between two operators on interaction with the *lac* repressor.. J Biol Chem.

[31] Krämer H, Amouyal M, Nordheim A, Müller-Hill B (1988). DNA supercoiling changes the spacing requirement of two *lac* operators for DNA loop formation with *lac* repressor.. EMBO J.

[32] Whitson PA, Hsieh WT, Wells RD, Matthews KS (1987). Inuence of supercoiling and sequence context on operator DNA binding with *lac* repressor.. J Biol Chem.

[33] Borowiec JA, Zhang L, Sasse-Dwight S, Gralla JD (1987). DNA supercoiling promotes formation of a bent repression loop in *lac* DNA.. J Mol Biol.

[34] Cloutier TE, Widom J (2005). DNA twisting exibility and the formation of sharply looped protein-DNA complexes.. Proc Natl Acad Sci U S A.

[35] Zhang Y, McEwen AE, Crothers DM, Levene SD (2006). Statistical-mechanical theory of DNA looping.. Biophys J.

[36] Swigon D, Coleman BD, Olson WK (2006). Modeling the Lac repressor-operator assembly: The inuence of DNA looping on Lac repressor conformation.. Proc Natl Acad Sci U S A.

[37] Geanacopoulos M, Vasmatzis G, Zhurkin VB, Adhya S (2001). Gal repressosome contains an antiparallel DNA loop.. Nat Struct Biol.

[38] Balaeff A, Mahadevan L, Schulten K (2006). Modeling DNA loops using the theory of elasticity.. Phys Rev E Stat Nonlin Soft Matter Phys.

[39] Towles K, Beausang JF, Garcia HG, Phillips R, Nelson PC (2009). First-principles calculation of DNA looping in tethered particle experiments.. Physical Biology, *in press*.

[40] Ackers GK, Johnson AD, Shea MA (1982). Quantitative model for gene regulation by lambda phage repressor.. Proc Natl Acad Sci U S A.

[41] Buchler NE, Gerland U, Hwa T (2003). On schemes of combinatorial transcription logic.. Proc Natl Acad Sci U S A.

[42] Bintu L, Buchler NE, Garcia HG, Gerland U, Hwa T (2005). Transcriptional regulation by the numbers: models.. Curr Opin Genet Dev.

[43] Bintu L, Buchler NE, Garcia HG, Gerland U, Hwa T (2005). Transcriptional regulation by the numbers: applications.. Curr Opin Genet Dev.

[44] Friedman AM, Fischmann TO, Steitz TA (1995). Crystal structure of *lac* repressor core tetramer and its implications for DNA looping.. Science.

[45] Mehta RA, Kahn JD (1999). Designed hyperstable Lac repressor. DNA loop topologies suggest alternative loop geometries.. J Mol Biol.

[46] Semsey S, Tolstorukov MY, Virnik K, Zhurkin VB, Adhya S (2004). DNA trajectory in the gal repressosome.. Genes Dev.

[47] Levandoski MM, Tsodikov OV, Frank DE, Melcher SE, Saecker RM (1996). Cooperative and anticooperative effects in binding of the first and second plasmid Osym operators to a LacI tetramer: evidence for contributions of non-operator DNA binding by wrapping and looping.. J Mol Biol.

[48] Barry JK, Matthews KS (1999). Thermodynamic analysis of unfolding and dissociation in lactose repressor protein.. Biochemistry.

[49] Saiz L, Rubi JM, Vilar JM (2005). Inferring the *in vivo* looping properties of DNA.. Proc Natl Acad Sci U S A.

[50] deHaseth PL, Gross CA, Burgess RR, Record MT (1977). Measurement of binding constants for protein-DNA interactions by DNA-cellulose chromatography.. Biochemistry.

[51] O'Gorman RB, Dunaway M, Matthews KS (1980). DNA binding characteristics of lactose repressor and the trypsin-resistant core repressor.. J Biol Chem.

[52] Revzin A, von Hippel PH (1977). Direct measurement of association constants for the binding of *escherichia coli lac* repressor to non-operator DNA.. Biochemistry.

[53] Record MT, deHaseth PL, Lohman TM (1977). Interpretation of monovalent and divalent cation effects on the *lac* repressor-operator interaction.. Biochemistry.

[54] Kao-Huang Y, Revzin A, Butler AP, O'Conner P, Noble DW (1977). Nonspecific DNA binding of genome-regulating proteins as a biological control mechanism: measurement of DNA-bound *escherichia coli lac* repressor *in vivo*.. Proc Natl Acad Sci U S A.

[55] Wang AC, Revzin A, Butler AP, von Hippel PH (1977). Binding of E. coli lac repressor to nonoperator DNA.. Nucleic Acids Res.

[56] Barkley MD (1981). Salt dependence of the kinetics of the *lac* repressor-operator interaction: role of nonoperator deoxyribonucleic acid in the association reaction.. Biochemistry.

[57] Zhang X, Gottlieb PA (1993). Thermodynamic and alkylation interference analysis of the *lac* repressor-operator substituted with the analogue 7-deazaguanine.. Biochemistry.

[58] Mossing MC, Record MT (1985). Thermodynamic origins of specificity in the *lac* repressoroperator interaction. adaptability in the recognition of mutant operator sites.. J Mol Biol.

[59] Horton N, Lewis M, Lu P (1997). *Escherichia coli lac* repressor-*lac* operator interaction and the inuence of allosteric effectors.. J Mol Biol.

[60] Goeddel DV, Yansura DG, Caruthers MH (1977). Binding of synthetic lactose operator DNAs to lactose represessors.. Proc Natl Acad Sci U S A.

[61] Falcon CM, Matthews KS (1999). Glycine insertion in the hinge region of lactose repressor protein alters DNA binding.. J Biol Chem.

[62] Winter RB, von Hippel PH (1981). Diffusion-driven mechanisms of protein translocation on nucleic acids. 2. The *escherichia coli* repressor–operator interaction: equilibrium measurements.. Biochemistry.

[63] Saiz L, Vilar JM (2006). DNA looping: the consequences and its control.. Curr Opin Struct Biol.

[64] Lee DH, Schleif RF (1989). *In vivo* DNA loops in araCBAD: size limits and helical repeat.. Proc Natl Acad Sci U S A.

[65] Law SM, Bellomy GR, Schlax PJ, Record MT (1993). *In vivo* thermodynamic analysis of repression with and without looping in *lac* constructs. estimates of free and local *lac* repressor concentrations and of physical properties of a region of supercoiled plasmid DNA *in vivo*.. J Mol Biol.

[66] Shore D, Baldwin RL (1983). Energetics of DNA twisting. I. Relation between twist and cyclization probability.. J Mol Biol.

[67] Du Q, Smith C, Shiffeldrim N, Vologodskaia M, Vologodskii A (2005). Cyclization of short DNA fragments and bending uctuations of the double helix.. Proc Natl Acad Sci U S A.

[68] Record MT, Mazur S, Melancon P, Roe JH, Shaner SL (1981). Double helical DNA: conformations, physical properties, and interactions with ligands.. Annual review of biochemistry.

[69] Strick T, Allemand J, Croquette V, Bensimon D (2000). Twisting and stretching single DNA molecules.. Progress in biophysics and molecular biology.

[70] Moroz JD, Nelson P (1998). Entropic elasticity of twist-storing polymers.. Macromolecules.

[71] Segall DE, Nelson PC, Phillips R (2006). Volume-exclusion effects in tethered-particle experiments: Bead size matters.. Physical Review Letters.

[72] Yan J, Marko JF (2004). Localized single-stranded bubble mechanism for cyclization of short double helix DNA.. Phys Rev Lett.

[73] Wiggins PA, Nelson PC, Phillips R (2005). Exact theory of kinkable elastic polymers.. Phys Rev E.

[74] Nelson PC, Zurla C, Brogioli D, Beausang JF, Finzi L (2006). Tethered particle motion as a diagnostic of DNA tether length.. J Phys Chem B.

[75] Czapla L, Swigon D, Olson WK (2006). Sequence-dependent effects in the cyclization of short DNA.. Journal of Chemical Theory and Computation.

[76] Nelson PC (2007). Colloidal particle motion as a diagnostic of DNA conformational transitions.. Curr Op Colloid Intef Sci.

[77] Lewis M, Chang G, Horton NC, Kercher MA, Pace HC (1996). Crystal structure of the lactose operon repressor and its complexes with DNA and inducer.. Science.

[78] Strick TR, Croquette V, Bensimon D (1998). Homologous pairing in stretched supercoiled DNA.. Proc Natl Acad Sci USA.

[79] Wang MD, Yin H, Landick R, Gelles J, Block SM (1997). Stretching DNA with optical tweezers.. Biophys J.

[80] Villa E, Balaeff A, Schulten K (2005). Structural dynamics of the *lac* repressor-DNA complex revealed by a multiscale simulation.. Proc Natl Acad Sci U S A.

[81] Wiggins PA, Van der Heijden T, Moreno-Herrero F, Spakowitz A, Phillips R (2006). High exibility of DNA on short length scales probed by atomic force microscopy.. Nature Nanotech.

[82] Ruben GC, Roos TB (1997). Conformation of *lac* repressor tetramer in solution, bound and unbound to operator DNA.. Microsc Res Tech.

[83] Edelman LM, Cheong R, Kahn JD (2003). Fluorescence resonance energy transfer over approximately 130 basepairs in hyperstable Lac repressor-DNA loops.. Biophys J.

[84] Morgan MA, Okamoto K, Kahn JD, English DS (2005). Single-molecule spectroscopic determination of Lac repressor-DNA loop conformation.. Biophys J.

[85] Zhang Y, McEwen AE, Crothers DM, Levene SD (2006). Analysis of *in-vivo* LacR-mediated gene repression based on the mechanics of DNA looping.. PLoS ONE.

[86] Oehler S, Amouyal M, Kolkhof P, von Wilcken-Bergmann B, Müller-Hill B (1994). Quality and position of the three *lac* operators of *E. coli* define efficiency of repression.. EMBO J.

[87] Lutz R, Bujard H (1997). Independent and tight regulation of transcriptional units in *Escherichia coli* via the LacR/O, the TetR/O and AraC/I1-I2 regulatory elements.. Nucleic Acids Res.

[88] Rosenfeld N, Young JW, Alon U, Swain PS, Elowitz MB (2005). Gene regulation at the single-cell level.. Science.

[89] Gelles J, Schnapp B, Sheetz M (1988). Tracking kinesin-driven movements with nanometre-scale precision.. Nature.

[90] Colquhoun D, Sigworth FJ, Sakmann B, Neher E (1995). Fitting and statistical analysis of single-channel records.. Single-Channel Recording.

[91] Motion of the bead is systematically characterized with various DNA lengths ranging from 200 bp to 3 kbp. Such DNA is then interpolated using a second order polynomial function to served as a calibration curve. From this curve, for any given length DNA tether in that range, the amplitude of the motion of the DNA tethered bead can be evaluated. Experimental data, and a theoretical model of the calibration, appear in [39]

[92] Frank DE, Saecker RM, Bond JP, Capp MW, Tsodikov OV (1997). Thermodynamics of the interactions of *lac* repressor with variants of the symmetric *lac* operator: effects of converting a consensus site to a non-specific site.. J Mol Biol.

[93] Oehler S, Eismann ER, Kramer H, Muller-Hill B (1990). The three operators of the *lac* operon cooperate in repression.. EMBO J.

