# Supplementary material for: Concentration and Length Dependence of DNA Looping in Transcriptional Regulation
Source: PLoS One. 2009 May 25;4(5):e5621. doi: 10.1371/journal.pone.0005621 (PMC2682762; doi:10.1371/journal.pone.0005621)
Supplement: Supporting Information S1 — (10.68 MB PDF) [file pone.0005621.s001.pdf]

Supporting Information S1: Concentration and Length Dependence of DNA Looping in Transcriptional Regulation

Lin Han[1], Hernan G. Garcia[2], Seth Blumberg[1], Kevin B. Towles[3], John F. Beausang[3], Philip C. Nelson[3], Rob Phillips[1,*]
1 Department of Applied Physics, California Institute of Technology, Pasadena CA 91125, USA
2 Department of Physics, California Institute of Technology, Pasadena CA 91125, USA
3 Department of Physics and Astronomy, University of Pennsylvania, Philadelphia PA 19104, USA
∗ **E-mail: phillips@pboc.caltech.edu**

S1 Bead Selection, Data Rejection and "Representative Data"

One of the most important challenges of these experiments (and perhaps any single-molecule experiment based upon watching the motions of beads tethered to single molecules) is devising systematic methods for deciding which beads are "qualified" and how to reject trajectories that are anomalous without biasing the results [1–4]. To that end, we have attempted to institute a number of criteria for performing data selection that are indicated schematically in figs. S1 and S2. The first attempt to "objectively" select qualified beads takes place by excising segments of the traces corresponding to the unlooped state and examining whether their motions are symmetric (i.e. jiggle in the x- and y- directions in the same way) as evidenced by the probability distribution for the x- and y- excursions. This screening permits us to select beads within a given field of view that are ostensibly properly tethered. Examples of these selection criteria are shown in fig. S1 for the particular case where no protein is present. Typically, a fraction of roughly 20 $\sim$ 30 % of the beads are rejected as a result of failure to exhibit proper symmetry or because they are stuck.

A more tricky question arises when we have to assess whether something went wrong during data acquisition that requires either all or part of a given TPM trajectory to be rejected. In some cases, the offending behavior is evident at the level of the bare images of the jiggling beads. For example, a given bead can become stuck to the surface or the DNA can break and the bead will disappear from the field of view. These events have a signature of spikes in the R_{RMS} traces as shown in fig. S2. A movie corresponding to the event shown in fig. S2(A) can be found as a Supplementary Movie.

Fig. S2 also shows an example of data that was kept with an offending region highlighted that was removed. Note that if the spike regions in trajectories were actually kept, it would have no bearing on histograms like those shown in figs. 2 and 7 since the spikes will show up as features on the tails of the histograms. On the other hand, by excising certain pieces of trajectories, there can be some effect on the kinetic claims we would be able to make since these anomalies will cause errors in the dwell time measurements.

In none of the cases considered in this work were sticking events observed in any significant number. Assuming that sticking is mainly due to nonspecific interactions with the bead and the surface one would expect the shorter constructs to show the most sticking events. In order to control for this we performed TPM experiments using tethers of 351 bp in length in the absence of Lac repressor. This length is comparable to the length the short constructs (E889, E894 and E8100) would have if the sequence between the *lac* operators was removed. Out of 18 tethers characterized only 5 showed any sticking events. In those 5 traces, the sticking events corresponded to less than 4 % of the observation time for each bead (data not shown). In order to discard any contribution to the sticking events from the presence of the protein, Lac repressor was flowed in in the presence of 1 mM IPTG which serves to eliminate the binding of Lac repressor to the DNA (or at least drastically reduce it). The goal of this control is to see whether the presence of unbound protein somehow induces unwanted sticking events. Out of the 7 tethers characterized all showed sticking events, but these corresponded to less than 1 %

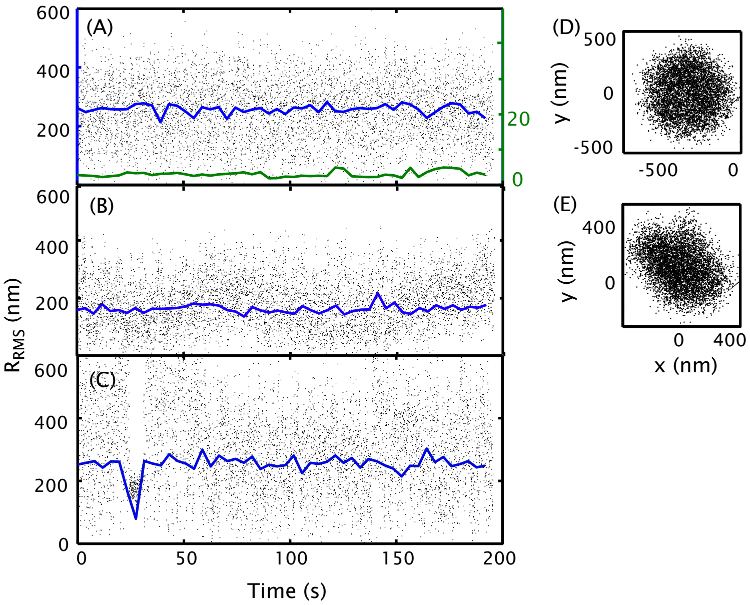

Figure S1. Conceptual description of data selection. All traces in this case are taken in the absence of Lac repressor and are used as the basis of choosing qualified beads for the looping study. (A) Experimental traces for a bead exercising symmetric motion (blue) and for a stuck bead (green). (B) Trajectory for a bead that exhibits non symmetric motion. (C) Trajectory for a bead that exhibits a transient sticking event. (D) Positional data for a bead that exhibits symmetric motion. (E) Positional data corresponding to the trajectory shown in (B) and for which the motion is not symmetric.

of the time. Finally, there is still the chance that Lac repressor that is specifically bound to the tether might contribute to sticking. In order to test this hypothesis we used a construct of this length with only one binding site. Here too (data not shown), there was no significant sticking lending further support for the idea that even for the short tethers, we are able to detect looping.

In order to produce histograms like those shown in figs. 2 and 7 we have to sum over the histograms resulting from many individual trajectories. Fig. 1 shows the connection between an individual TPM trace for a single bead and its corresponding motion histogram. However, since each trajectory has its own unique features, it is of interest to see how the smoothed histogram resulting from many individual trajectories emerges from the averaging process. Fig. S3 shows the motion histogram obtained by averaging over the histograms from progressively larger numbers of individual trajectories.

Now that we have seen some of the pitfalls associated with TPM trajectories, we show "representative" examples of the individual trajectories culminating in figs. 2 and 7. Fig. S4 shows multiple examples of trajectories resulting from different concentrations of Lac repressor. Even at the level of visual inspection of these individual trajectories, it is evident that there are two distinct looping states and that the relative occupancies of the different looped and unlooped states depend upon the concentration of repressor. Similar results are shown in figs. S5 and S6 which illustrates multiple individual trajectories for the case in which the interoperator spacing (rather than the Lac repressor) concentration is the experimental dial that we tune to vary the looping stability.

S2 Data Analysis and Probabilities Calculation

The data shown in figs. 2 and 7 characterizes the results of many different TPM trajectories for each condition (Lac repressor concentration or interoperator spacing). We are interested in obtaining the probabilities associated with each state and to that end, we have tried a variety of different approaches to examine the sensitivity of the results to method of data analysis.

The first analysis we explored is based on directly looking at histograms such as those shown in figs. 2 and 7. As mentioned in the main text, these histograms are the result of adding up the normalized contribution from each bead. One scheme for carrying this out is to fit the histogram to the sum of three Gaussians. The idea of such a fit is that there is a main peak associated with the unlooped state and then two separate looping peaks, each of which is fit with its own Gaussian. With the fitting results in hand the area under each Gaussian can be computed, which leads to a probability assignment. We call this scheme "Gaussian Integral".

An alternative scheme is to define thresholds between the different states. The bins on either side of the thresholds are then added, giving the different probabilities. We explore two ways of calculating the thresholds: i) Finding the minimum between adjacent Gaussians from the fit described previously ("Gaussian Minimum"), and ii) finding the minimum in the histograms between peaks ("Histogram Minimum").

Finally, we have also explored the use of alternatives such as the Diffusive Hidden Markov Model ("dHMM"). The Diffusive Hidden Markov Model (DHMM) method is applied to do the kinetic analysis [5, 6] and for our present purposes permits us a different way to determine the looping probability by telling us the fraction of time spent in each of the distinct states. This method employs the concept of HMM and customizes it in a way suitable for TPM data, through which the rate constants are directly derived from the positional data obtained in the TPM experiments. To characterize the dynamical information of the beads in each state, control experiments are performed in the following ways: i) To obtain the information for the unlooped state, the bead's motion is observed in the absence of the DNA looping protein Lac repressor. ii) For the looped state, we monitor the bead's motion in the presence of a Lac repressor mutant V52C instead of Lac repressor itself. This mutant is designed to permit disulfide bond formation, which makes important contacts that are critical to DNA binding. As a result, V52C has increased affinity for DNA operators [7], leading to a measurement of primarily looped states. Such data

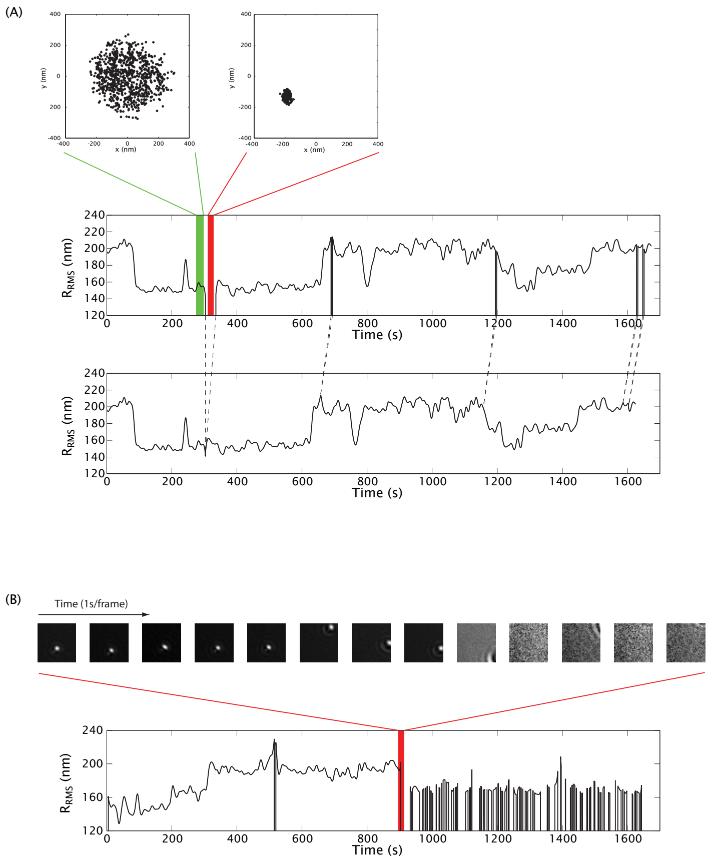

Figure S2. Transient sticking events and tether breaking. (A) A transient sticking event is revealed by a dramatic reduction in the movement of the bead and is associated with a spike in the R_{RMS} trace. These "offending" regions of the traces can be excised out which will not affect the resulting histogram, but might present an issue for any kinetic analysis as discussed in the text. A movie corresponding to this event is provided as a Supplementary Movie. (B) Signature of a tether breaking. Each movie frame is rescaled based on its maximum and minimum pixel value, which leads to overall differences in intensity between frames.

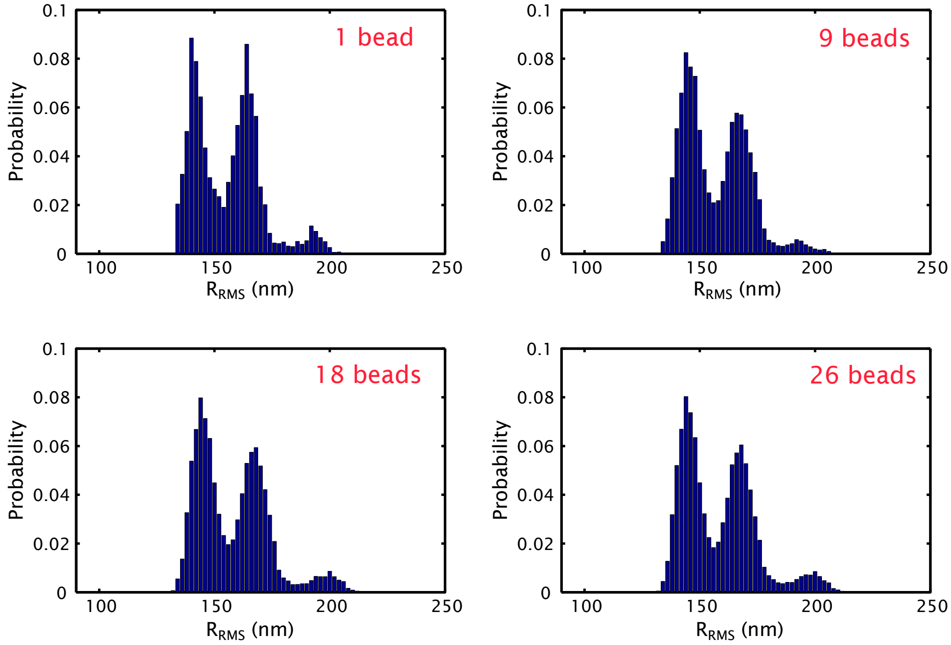

Figure S3. Effect of averaging on the data. These four histograms show the effects of including different numbers of beads in determining the overall average. Data obtained with pUC306L1 DNA in the presence of 10 pM Lac repressor.

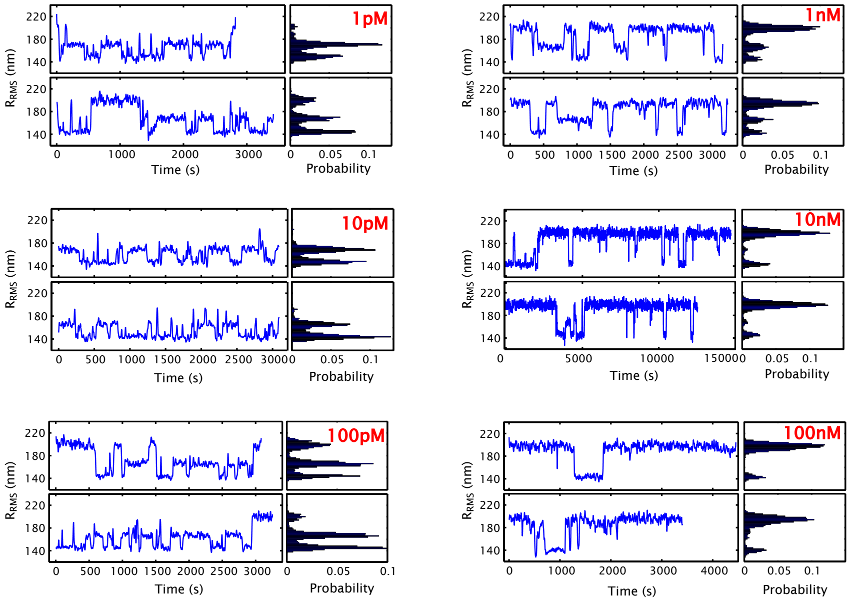

Figure S4. Concentration dependence of TPM trajectories. Representative examples of TPM trajectories. Typical TPM trajectories of the DNA tethered beads in the presence of different concentrations of Lac repressor varying from 1 pM to 100 nM. The total DNA length is 901 bp and the interoperator spacing is 306 bp.

containing only one type of looped state is selected to obtain the information that serves as input to the HMM model. One of the outcomes of the HMM analysis is an explicit statement about the amount of time spent in each of the states which can be used in turn to compute the looping probability.

One argument against the previously mentioned schemes is that they do not capture the variability inherent in single molecule experiments. Each tether will behave in a slightly different way, as is illustrated in fig. S7 for construct pUC300L1. Notice that even though the two looped states were overlapping in fig. 7 they are discernable in most individual traces. Fig. S7(F) also shows a case where no call on the identity of the looped state could be made. For the long length constructs where this happened only a small fraction of the beads, between 2% and 6% would show this type of histogram. Identification of the individual loops becomes more problematic in the short length constructs. In this case around 10% of the beads would show this behavior.

The looping probabilities obtained using all these methods are shown in fig. S8. We conclude that there is no significant variation in the results from any of the different approaches. In section S4 we show that the quantitative parameters extracted from these different looping probabilities do not differ significantly. Finally, figs. S9 and S10 show the looping probability for each individual state in the cases where both states were discernible. Ultimately, it would be of great interest to use experiments like those described here to determine the looping free energies (or J_{loop}s for the different states. This is presented in section S5.

S3 Theoretical Analysis of Looping

Statistical mechanics provides a powerful tool for dissecting the DNA-protein interactions that take place during transcriptional regulation. We find it convenient to derive the various expressions for binding

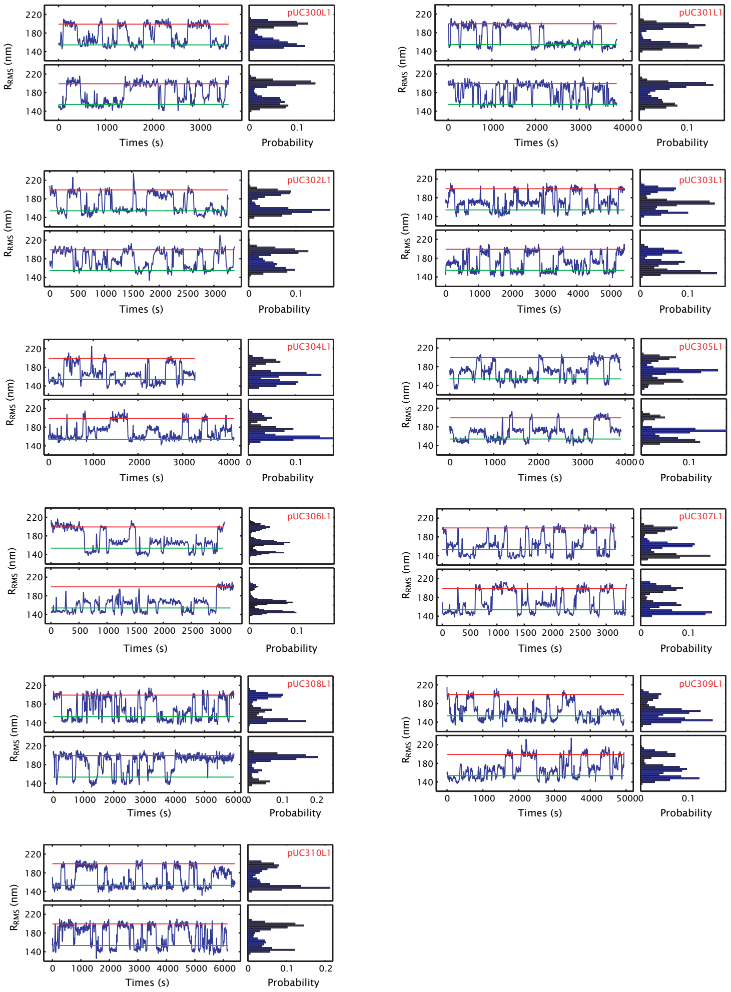

Figure S5. Length dependence of TPM trajectories. Typical TPM trajectories of the DNA tethered beads with interoperator spacing from 300 to 310 bp in 1 bp increments. The concentration of Lac repressor used in this set of experiments was 100 pM. The distance between the two operators is indicated in the naming of the construct.

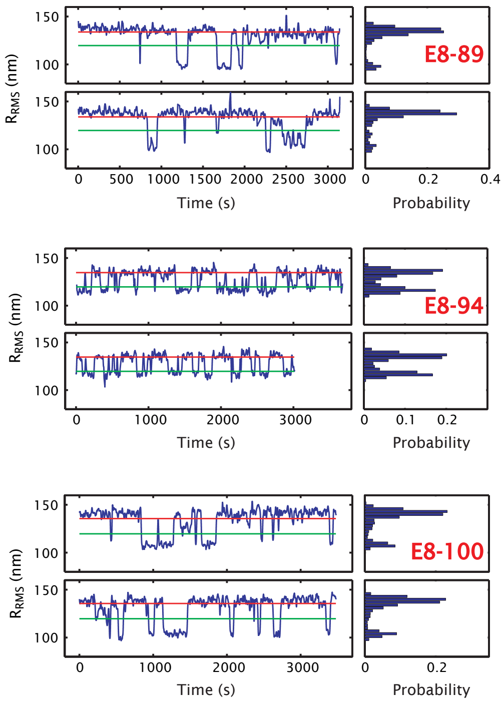

Figure S6. Typical TPM trajectories for DNA tethered beads with interoperator spacing of 89 bp, 94 bp and 100 bp. E8 refers to the particular sequence used in these experiments. The concentration of Lac repressor used to generate these trajectories is 100pM. The red and green lines indicate the expected excursion for the unlooped and looped states, respectively, where the expected length of the looped state is based upon subtracting the interoperator spacing from the overall tether length.

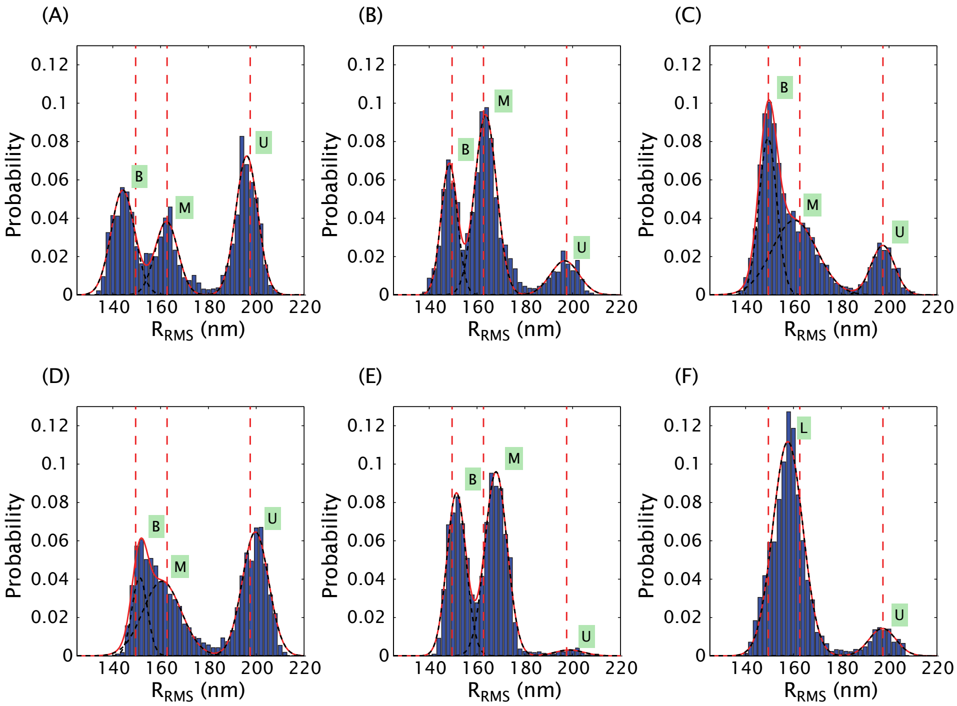

Figure S7. Rogues gallery of individual bead histograms. Three Gaussian fit to individual bead traces corresponding to the pUC300L1 construct. The vertical dashed lines correspond to the locations of the peaks as revealed by a three Gaussian fit to the corresponding histogram of fig. 7. The black dashed line are the individual Gaussians, while the solid red line is their sum. (A-E) The peaks are labeled B (bottom loop), M (middle loop), and U (unlooped state). In the small fraction of cases that no decision about the identity of the looped state could be made the label L (looped state) is used.

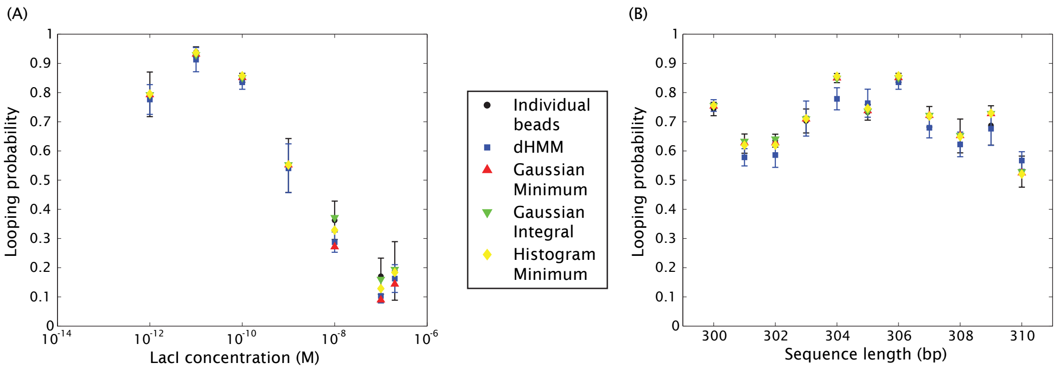

Figure S8. Different approaches for calculating the looping probability. The looping probability as a function of (A) concentration and (B) sequence length, calculated using the approaches described in the text.

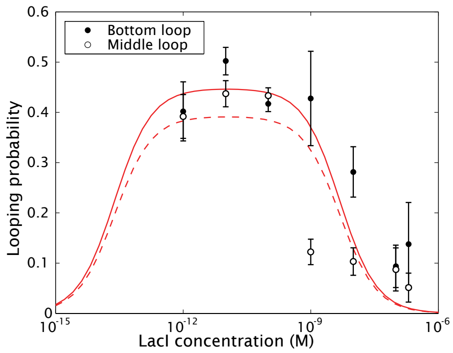

Figure S9. Individual loops vs. concentration. Probability of each looped state as a function of concentration. The lines are fits to the nonlinear model from eqn. S14.

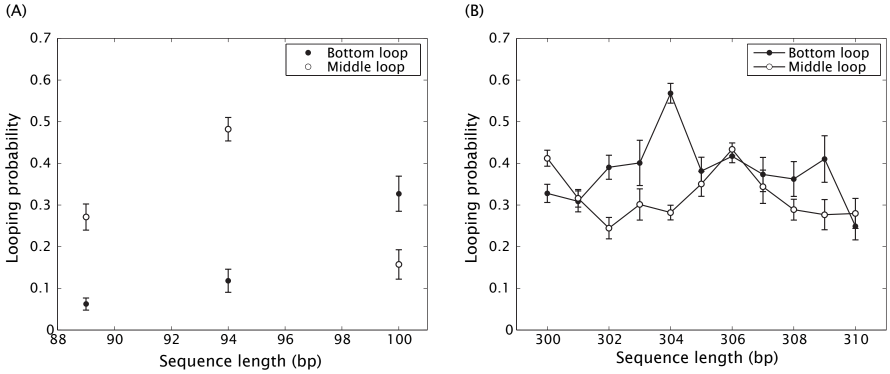

Figure S10. Individual loops vs. phasing. Probability of each looped state as a function of sequence length. (A) Short loops, (B) a full cycle at 300 bp.

probabilities using simple lattice models of DNA binding proteins and their DNA targets. These models can then be reinterpreted in the familiar language of equilibrium constants and effective J-factors. In this section, we sketch the derivations of the formulae used in the main body of the paper. An alternative derivation appears in [8].

S3.1 Simple binding of Lac repressor

In a lattice model, we imagine the solution as discretized into a set of Ω boxes of volume v. The R repressors are free to occupy any of these distinct boxes which provide a simple and convenient basis for computing the entropic contribution to the overall free energy. A repressor in solution has an energy ε_{sol} which appears in the Boltzmann factor. The configurational degrees of freedom (both translational and rotational) in this model are taken care of by assigning the molecules to one of the Ω boxes available in our lattice model of the solution and by noting that there is a factor of $\frac{8\pi^2}{\delta\omega}$ associated with its rotational degrees of freedom (4π for the directions in which the molecule can point on the unit sphere and 2π for the rotation around the protein's axis). The partition function of R repressors in the solution is

$$Z_{sol} = \binom{\Omega}{R} e^{-\beta R \varepsilon_{sol}} \left(\frac{8\pi^2}{\delta\omega}\right)^R. \tag{S1}$$

Now we introduce a DNA molecule with one binding site. This case is appropriate when LacI is in excess of the DNA. When one Lac repressor from the solution binds to the operator it now has an energy ε_b associated with the binding itself and a "tether" energy ε_t associated with the extra binding head that is still in the solution. Next, we exploit the fact that we can choose either head to bind to the operator of interest and this head can bind in two distinct orientations, yielding a factor of 4 degeneracy in this state. The total partition function is

$$Z = Z_{sol}(R) + 4Z_{sol}(R-1)e^{-\beta(\varepsilon_b+\varepsilon_t)}. \tag{S2}$$

This translates into the following probability of binding

$$p_{bound} = \frac{4\frac{\delta\omega}{8\pi^2}\frac{R}{\Omega}e^{-\beta\Delta\varepsilon}}{1+4\frac{\delta\omega}{8\pi^2}\frac{R}{\Omega}e^{-\beta\Delta\varepsilon}}, \tag{S3}$$

where we have defined $\Delta\varepsilon = \varepsilon_b + \varepsilon_t - \varepsilon_{sol}$.

We recover the usual formula when characterizing binding using dissociation constants

$$p_{bound} = \frac{[R]/K_d}{1+[R]/K_d}, \tag{S4}$$

if we make the identification

$$K_d = \frac{1}{4v}\frac{8\pi^2}{\delta\omega}e^{\beta\Delta\varepsilon}. \tag{S5}$$

With this result in hand we are ready to address the more complex case of DNA looping.

S3.2 DNA looping by Lac repressor

We now have two operators, each one with a binding energy ε_1 and ε_{id}, corresponding to the operators $O1$ and Oid, respectively. We consider the usual five classes of states that include: i) free operators, ii+iii) one of the operators occupied, iv) both operators occupied by different LacI molecules, and v) LacI

looping both operators, which can happen in multiple configurations. The partition function is

$$\begin{aligned} Z &= Z_{sol}(R) + 4Z_{sol}(R-1)e^{-\beta\varepsilon_t}\left(e^{-\beta\varepsilon_1} + e^{-\beta\varepsilon_{id}}\right) \\ &\quad +16Z_{sol}(R-2)e^{-\beta(\varepsilon_1+\varepsilon_{id}+2\varepsilon_t)} + \\ &\quad +\sum_i Z_{sol}(R-1)e^{-\beta(\varepsilon_1+\varepsilon_{id}+F_{loop,i})}. \end{aligned} \tag{S6}$$

The factors of 4 in the second and third term correspond to the degeneracy described above. The factor of 16 in the fourth term accounts for all of the different ways of binding two repressors independently. Here we defined $F_{loop,i}$ as the looping free energy associated with a particular configuration (orientation of operators with respect to the molecule). The sum in the last term includes all four possible loop topologies [9,10] and the fact that we are thinking of the two binding heads of LacI as being distinguishable. Defining α and β as state variables that describe the orientation of $O1$ and Oid with respect to the binding heads (see fig. 5, respectively we can write the sum as

$$\sum_i = \sum_{\text{heads}} \sum_{\alpha,\beta}. \tag{S7}$$

The sum over the heads results in a factor of two, since none of the terms inside the sum actually depend on that choice. We next define the overall looping energy ΔF_{loop} by

$$e^{-\beta\Delta F_{\text{loop}}} = \frac{1}{\sum_{\alpha,\beta} 1}\sum_{\alpha,\beta} e^{-\beta F_{\text{loop},\alpha,\beta}} = \frac{1}{4}\sum_{\alpha,\beta} e^{-\beta\Delta F_{\text{loop},\alpha,\beta}}. \tag{S8}$$

Using the calculations and definitions from section S3.1 we arrive at the looping probability

$$\begin{aligned} p_{\text{loop}} &= \left[8\frac{R}{\Omega}\frac{\delta\omega}{8\pi^2}e^{-\beta(\Delta\varepsilon_1+\Delta\varepsilon_{id}+\Delta F_{\text{loop}}+2\varepsilon_t-\varepsilon_{sol})}\right] \\ &\quad \left[1 + 4\frac{R}{\Omega}\frac{\delta\omega}{8\pi^2}\left(e^{-\beta\Delta\varepsilon_1} + e^{-\beta\Delta\varepsilon_{id}}\right) + 16\frac{R(R-1)}{\Omega^2}\left(\frac{\delta\omega}{8\pi^2}\right)^2 e^{-\beta(\Delta\varepsilon_1+\Delta\varepsilon_{id})} + \right. \\ &\quad \left. 8\frac{R}{\Omega}\frac{\delta\omega}{8\pi^2}e^{-\beta(\Delta\varepsilon_1+\Delta\varepsilon_{id}+\Delta F_{\text{loop}}+2\varepsilon_t-\varepsilon_{sol})}\right]^{-1}. \end{aligned} \tag{S9}$$

Notice that the term that corresponds to looping has the energy $\Delta F_{\text{loop}} + 2\varepsilon_t - \varepsilon_{sol}$. In principle this is the parameter associated with looping, but it also includes information about the energetics of LacI when it is in solution and when it has only one head bound to the DNA. However, we can make the assumption that the energy associated with having half a LacI in solution, ε_t is half the energy of having a full LacI in solution, ε_{sol}. This is equivalent to saying that there is no change in the energetics of binding if the other head is already bound, that there is no allosteric cooperativity. If this is true then the parameter obtained from an experiment where p_{loop} is measured will actually be ΔF_{loop}.

Since we measure concentration of Lac repressor rather than absolute number of repressor molecules we want to rewrite this formula as a function of $[R]$ using the lattice definitions

$$\frac{R}{\Omega} = \frac{R}{\Omega v}v = [R]v. \tag{S10}$$

The parameter v corresponds to the volume of a lattice site, which means that Ωv corresponds to the whole volume. We now make the choice of a standard concentration

$$\frac{1}{v}\frac{8\pi^2}{\delta\omega} = 1 \text{ M}, \tag{S11}$$

which turns the looping probability from eqn. S9 into eqn. 1 which we repeat here for completeness

$$\begin{aligned} p_{\text{loop}} &= \left[8\frac{[R]}{1\text{ M}}e^{-\beta(\Delta\varepsilon_1+\Delta\varepsilon_{id}+\Delta F_{\text{loop}})}\right] \\ &\quad \left[1+4\frac{[R]}{1\text{ M}}\left(e^{-\beta\Delta\varepsilon_1}+e^{-\beta\Delta\varepsilon_2}\right)+16\left(\frac{[R]}{1\text{ M}}\right)^2 e^{-\beta(\Delta\varepsilon_1+\Delta\varepsilon_{id})}+\right. \\ &\quad \left. 8\frac{[R]}{1\text{ M}}e^{-\beta(\Delta\varepsilon_1+\Delta\varepsilon_{id}+\Delta F_{\text{loop}})}\right]^{-1}. \end{aligned}$$

Finally, we make the connection to the thermodynamic formalism using eqns. S5 and by defining that

$$J_{\text{loop}} = \frac{1}{v}\frac{8\pi^2}{\delta\omega}e^{-\beta\Delta F_{\text{loop}}}. \tag{S12}$$

The point here is to use simple binding to define the parameters K_1, K_{id} and cyclization to assign the parameter J_{loop} [11]. Here, we use a looping J_{loop} factor rather than the regular factor J factor to emphasize the fact that the boundary conditions are different from those present in cyclization, where J is clearly defined [12]. In this way, we appeal to these other experiments semantically and plug their definitions into the expression for the looping probability derived above. This results in eqn. 2, namely

$$p_{\text{loop}} = \frac{\frac{1}{2}\frac{[R]J_{\text{loop}}}{K_1K_{id}}}{1+\frac{[R]}{K_1}+\frac{[R]}{K_{id}}+\frac{[R]^2}{K_1K_{id}}+\frac{1}{2}\frac{[R]J_{\text{loop}}}{K_1K_{id}}},$$

where J_{loop} is the average of the individual J_{loop} factors over α and β as defined in eqn. 3.

In the case where we distinguish between bottom and middle looped states we can split J_{loop} into their corresponding looping J factors

$$J_{\text{loop}} = \frac{1}{2}\left(J_{\text{loop,B}}+J_{\text{loop,M}}\right). \tag{S13}$$

In this case, for example, the probability of looping into the bottom state can be written as

$$p_{\text{loop}} = \frac{\frac{1}{4}\frac{[R]J_{\text{loop,B}}}{K_1K_{id}}}{1+\frac{[R]}{K_1}+\frac{[R]}{K_{id}}+\frac{[R]^2}{K_1K_{id}}+\frac{1}{2}\frac{[R]J_{\text{loop}}}{K_1K_{id}}}. \tag{S14}$$

S4 Comparison of Theory and Experiment

One of the important goals of this work is to demand a rich interplay between theories of transcriptional regulation and corresponding experiments. To that end, the entirety of the data presented in the paper is viewed through the prism of the statistical mechanics model described above.

One of the questions that we have examined is how the statistical mechanics fit depends upon the choice of how we analyze the data to determine the looping probability. Examples of different schemes for determining the looping probability and their allied fits are shown in fig. S11. In the main body of the paper, we presented looping probabilities based upon Gaussian fits to the looping peaks. However, we have also explored the use of alternatives such as the Diffusive Hidden Markov Model.

Another point of curiosity concerns the extent to which our fits for the equilibrium constants and effective J-factor depends upon which points from fig. 3 are actually used to make the fit. Fig. S12 shows the fit to both K_1 and J_{loop} as a function of the particular model (nonlinear or linear) and range of data points from fig. 3 that are used in the fit. The key observation is that the final two data points (i.e. those

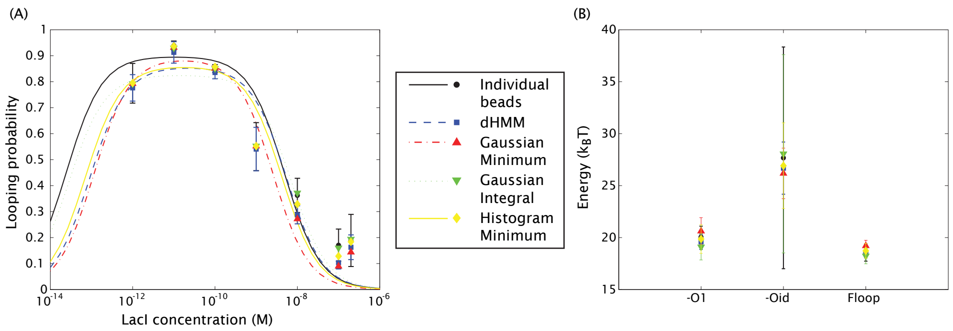

Figure S11. Alternative methods for fitting the looping probabilities. (A) Different schemes for determining the looping probability from the data result in slightly different fits for the concentration dependent data. (B) Results of the various fits performed in (A). Notice how the model cannot constrain the binding energy of *Oid* very accurately.

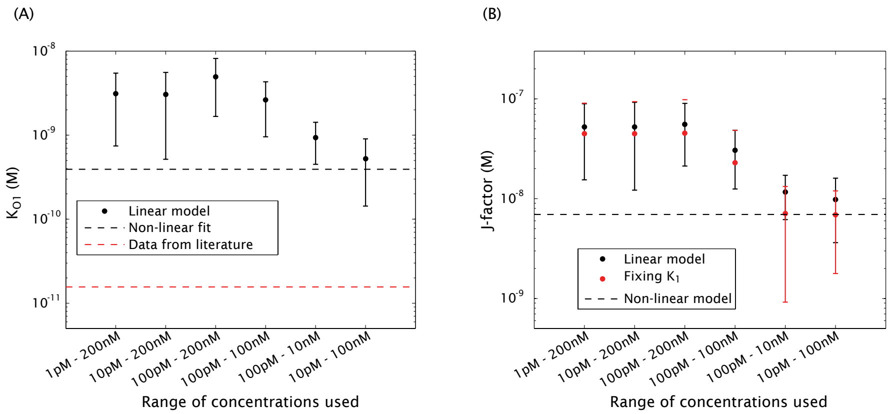

Figure S12. Sensitivity of fits to the method of data analysis. (A) Different fits to the value of K_1 using the linear model of eqn. 4 and different ranges of data points from fig. 3. The results corresponding to the non-linear model of eqn. 2 are also shown. (B) Different fits to the value of J_{loop} using the linear and non-linear models as shown in (A). "Fixing K_1" corresponds to fixing the $O1$ dissociation constant to the literature value shown in table 1.

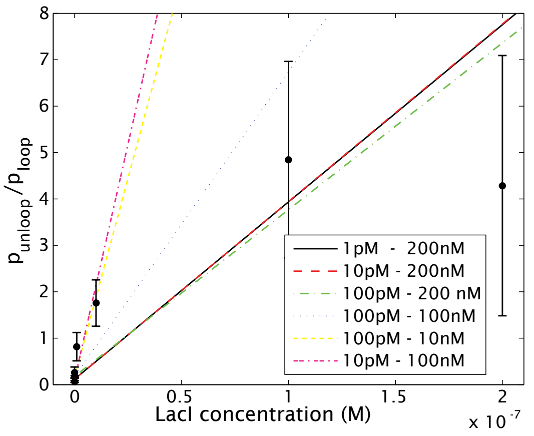

Figure S13. Sensitivity of linear fits to the range of data used. Different ranges of concentration from fig. 3 are fit using the linear model of eqn. 4.

at the largest concentrations of Lac repressor) lead to a systematic shift in the values for both K_1 and $J_{\rm loop}$ when fitting using the linear model from eqn. 4. Another interesting point revealed by fig. S12(A) is that the full nonlinear model fit results in a value for K_1 that is too large relative to the literature value by roughly a factor of 10, corresponding to a difference in binding energy of roughly 2 k_BT.

The dependence of our fits on the choice of data points included is also revealed in fig. S13. In this case, we show the result of using eqn. 4 as the basis of the fit and including different subsets of the data from fig. 3.

S5 Individual Looped States

In figs. S9 and S10 we showed the looping probabilities corresponding to each individual loop: the bottom and middle loops. In order to analyze these results we can construct an individual loop ratio analogous to the one defined in eqn. 4. For the case of the bottom loop, for example, this is

$$p_{\rm ratio,B} = \frac{p_{\rm loop,B}}{p_{\rm unloop}} = \frac{4K_1}{J_{\rm loop,B}} + \frac{4[R]}{J_{\rm loop,B}}. \qquad \text{(S15)}$$

Using an approach analogous to the one leading to eqn. 5 we obtain the looping J factors associated with each individual loop as shown in fig. S14. In fig. S15 we show their corresponding looping energies.

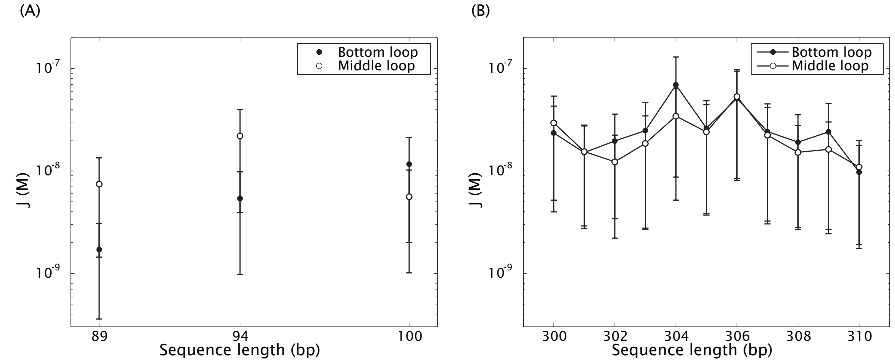

Figure S14. Individual loops J_{loop} as a function of sequence length. (A) Results for short constructs, (B) results for long constructs.

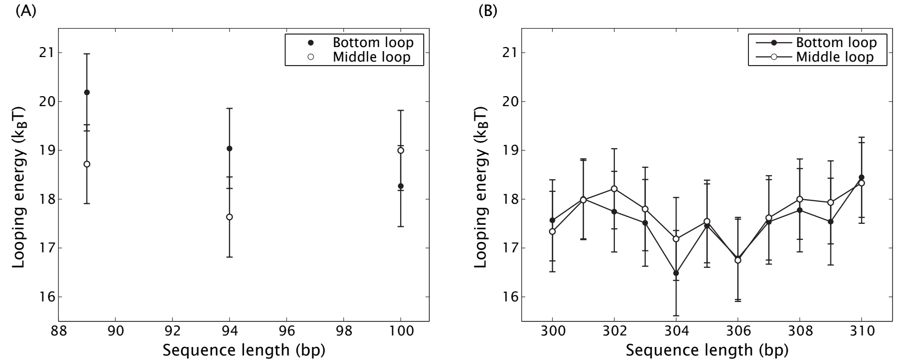

Figure S15. Individual loops energies as a function of sequence length. (A) Results for short constructs, (B) results for long constructs.

S6 Monte Carlo simulation

Our mathematical model built on our previous work [13–15], which showed that a Gaussian-sampling simulation could accurately model the experimentally observed relation between DNA tether length and TPM bead motion by including an effective entropic stretching force from bead–wall repulsion. This technique is essentially a Monte Carlo evaluation of the equilibrium partition function of a chain. Instead of a Metropolis implementation, we simply generated many discretized chains using Gaussian distributions for each link's bending and twisting angles, then discarded any such chains that violated the global steric constraints. To compute looping J factors, we modified our previous code to monitor the separation and relative orientation of the operator centers in the generated chains, and found the fraction of all chains that met the conditions needed for looping. See [8] for more details.

To obtain the distributions of bead excursion shown in fig. 11, we needed to make a correction before comparing to the experimental data. Our video camera gathers light for almost the entire 33 ms video frame time. This time scale is an appreciable fraction of the bead's diffusion time in the trap created by its tether, leading to a blurring of the bead image and an apparent reduction of bead RMS excursion. We measured this effect by looking at the apparent RMS excursion for a bead/tether system with many different shutter times, then corrected our numerically generated values for the position of the bead center to account for blurring [8].

In addition, we reduced our simulation data in a way that parallels what was done with the experimental data. The experiment takes data in the form of a time series for the projected location of the bead center (relative to its attachment), that is, $(x(t), y(t))$. We found the length-squared of these position vectors, R^2, then applied a Gaussian filter that essentially averaged over a 4-s window. To simulate equilibrium averages in this context, we harvested batches of N_{samp} independent simulated chains and found the standard deviation of excursion within each batch. From the resulting series of values for $R_{\mathrm{RMS}} = \sqrt{\langle R^2 \rangle_{N_{\mathrm{samp}}}}$, we made a histogram representing the probability density function of R_{RMS}. To choose an appropriate value for N_{samp}, we found a characteristic time scale for bead diffusion from the time autocorrelation function of R_{RMS}, then divided the 4 s window into N_{samp} slots corresponding to the larger of the frame time, 33 ms, or the bead diffusion time [8].

References

1. Pouget N, Dennis C, Turlan C, Grigoriev M, Chandler M, et al. (2004) Single-particle tracking for DNA tether length monitoring. Nucleic Acids Res 32: e73.

2. Blumberg S, Tkachenko AV, Meiners JC (2005) Disruption of protein-mediated DNA looping by tension in the substrate DNA. Biophys J 88: 1692-701.

3. Pouget N, Turlan C, Destainville N, Salome L, Chandler M (2006) Is911 transpososome assembly as analysed by tethered particle motion. Nucleic Acids Res 34: 4313-23.

4. Nelson PC, Zurla C, Brogioli D, Beausang JF, Finzi L, et al. (2006) Tethered particle motion as a diagnostic of DNA tether length. J Phys Chem B 110: 17260-7.

5. Beausang JF, Zurla C, Manzo C, Dunlap D, Finzi L, et al. (2007) DNA looping kinetics analyzed using diffusive hidden Markov model. Biophysical journal 92: L64-6.

6. Beausang JF, Nelson PC (2007) Diffusive hidden Markov model characterization of DNA looping dynamics in tethered particle experiments. Physical Biology 4: 205–219.

7. Falcon CM, Swint-Kruse L, Matthews KS (1997) Designed disulfide between N-terminal domains of lactose repressor disrupts allosteric linkage. The Journal of biological chemistry 272: 26818-21.

8. Towles K, Beausang JF, Garcia HG, Phillips R, Nelson PC (2009) First-principles calculation of DNA looping in tethered particle experiments. Physical Biology, *in press* .

9. Semsey S, Tolstorukov MY, Virnik K, Zhurkin VB, Adhya S (2004) DNA trajectory in the gal repressosome. Genes Dev 18: 1898-907.

10. Swigon D, Coleman BD, Olson WK (2006) Modeling the Lac repressor-operator assembly: The influence of DNA looping on Lac repressor conformation. Proc Natl Acad Sci U S A 103: 9879-84.

11. Phillips R, Kondev J, Theriot J (2008) Physical Biology of the Cell, *in press*. New York: Garland Science.

12. Shore D, Langowski J, Baldwin RL (1981) DNA flexibility studied by covalent closure of short fragments into circles. Proc Natl Acad Sci U S A 78: 4833-7.

13. Segall DE, Nelson PC, Phillips R (2006) Volume-exclusion effects in tethered-particle experiments: Bead size matters. Physical Review Letters 96: 088306-(1–4).

14. Nelson PC, Zurla C, Brogioli D, Beausang JF, Finzi L, et al. (2006) Tethered particle motion as a diagnostic of DNA tether length. Journal of Physical Chemistry B 110: 17260-17267.

15. Nelson PC (2007) Colloidal particle motion as a diagnostic of DNA conformational transitions. Curr Op Colloid Intef Sci 12: 307–313.
